# Ortho-methylated 3-hydroxypyridines hinder hen egg-white lysozyme fibrillogenesis

**DOI:** 10.1038/srep12052

**Published:** 2015-07-14

**Authors:** Laura Mariño, Kris Pauwels, Rodrigo Casasnovas, Pilar Sanchis, Bartolomé Vilanova, Francisco Muñoz, Josefa Donoso, Miquel Adrover

**Affiliations:** 1 Institut Universitari d’Investigació en Ciències de la Salut (IUNICS). Departament de Química, Universitat de les Illes Balears, Ctra. Valldemossa km 7.5, E-07122 Palma de Mallorca, Spain; 2 Structural Biology Brussels, Vrije Universiteit Brussel, Pleinlaan 2, 1050 Brussel, Belgium; 3 VIB Structural Biology Research Centre, Vlaams Instituut voor Biotechnologie, Pleinlaan 2, 1050 Brussel, Belgium

## Abstract

Protein aggregation with the concomitant formation of amyloid fibrils is related to several neurodegenerative diseases, but also to non-neuropathic amyloidogenic diseases and non-neurophatic systemic amyloidosis. Lysozyme is the protein involved in the latter, and it is widely used as a model system to study the mechanisms underlying fibril formation and its inhibition. Several phenolic compounds have been reported as inhibitors of fibril formation. However, the anti-aggregating capacity of other heteroaromatic compounds has not been studied in any depth. We have screened the capacity of eleven different hydroxypyridines to affect the acid-induced fibrillization of hen lysozyme. Although most of the tested hydroxypyridines alter the fibrillation kinetics of HEWL, only 3-hydroxy-2-methylpyridine, 3-hydroxy-6-methylpyridine and 3-hydroxy-2,6-dimethylpyridine completely abolish fibril formation. Different biophysical techniques and several theoretical approaches are combined to elucidate their mechanism of action. *O*-methylated 3-hydroxypyridines bind non-cooperatively to two distinct but amyloidogenic regions of monomeric lysozyme. This stabilises the protein structure, as evidenced by enhanced thermal stability, and results in the inhibition of the conformational transition that precedes fibril assembly. Our results point to *o*-methylated 3-hydroxypyridines as a promising molecular scaffold for the future development of novel fibrillization inhibitors.

Many proteins, of diverse structure and function, can self-assemble into morphologically similar fibrillar aggregates that are commonly termed amyloid fibrils. Amyloid fibril deposition is associated with protein-misfolding disorders including prevalent neurodegenerative diseases such as Alzheimer’s, Parkinson’s, Huntington’s or prion diseases^1^. Functionally, amyloid fibrils are implicated in numerous physiological processes such as epigenetic inheritance, long-term memory or melanosome biogenesis^2^.

The assembly of amyloid fibrils is accompanied by structural changes in the aggregating proteins^3^. Their native functional state typically converts into a β-sheet-rich conformation^4^ suggesting that β-sheet formation drives the amyloid-assembly. This was supported by molecular dynamics simulations demonstrating that the increased stability of β-conformers favors fibrillogenesis^5^. Additionally, X-ray fiber diffraction showed that amyloid fibrils consist of a generic hydrogen-bonded β-sheet structure in which the β-strands run perpendicular to the long fiber axis^4^. The finding that amyloid fibrils are mainly stabilized by hydrogen bonds involving the peptide backbone explains why fibrils can be formed from different polypeptides while displaying similar ultrastructure and morphology.

Amyloid formation mechanism cannot always be described by simple nucleation polymerization models as some proteins seem to fibrillize through a non-nucleated process^6^, but also because different species have been identified along the aggregation pathways. Spherical oligomers and worm-like structures (also called ‘protofibrils’) have been proposed as crucial intermediates that hold the amyloidogenic toxicity^7^, in contrast to the apparently harmless mature fibrils. Therefore, the description of the structural determinants that drive these ‘pre-fibrillar species’ towards toxicity is of paramount importance, although it is still unclear. Some results suggest that the oligomeric cytotoxicity is inversely proportional to their size^8^. Alternatively, it has also been hypothesized that the oligomeric toxicity is related to the degree of solvent exposure of their hydrophobic regions^9^ but also to their flexibility and dynamics^10^.

The formation of amyloid fibrils and their transient ‘pre-fibrillar species’ has also been reported for proteins that are not (yet) directly disease-related, such as hen egg-white lysozyme (HEWL)^11^. Experimental evidence has shown that their corresponding oligomers have similar cytotoxicity to those obtained from proteins implicated in amyloidogenic diseases^12^. Hence, it becomes clear that the cytotoxicity of the aggregated species is held in its own architecture, and it is independent of the protein nature.

HEWL is a monomeric globular enzyme that can hydrolyze the bacterial cell wall^13^. Structurally, it is a helix-rich protein possessing a high degree of sequence similarity and structural homology to the human variant^14^, which is associated with the familial systemic amyloidosis^15^. HEWL is prone to aggregate in an acid environment at elevated temperature^16^, in presence of chemical denaturants^17^, under agitation^18^, or as a result of its non-enzymatic glycation^19^. Therefore, HEWL has become an appealing model to study the protein misfolding and amyloid fibril formation mechanisms^11^.

Intense efforts have been directed towards identifying or designing compounds that are able to counteract fibrillogenesis through: i) the stabilization of the native state, ii) the sequestration of monomeric peptide, iii) the stabilization or promotion of off-pathway oligomers, iv) the breakage of the β-sheet terminating the fibril elongation, or v) fibril disassembly^20^. Ruthenium complexes^21^, cysteine^22^ or 2,5-diarylated thiophenes^23^ have recently been found to disassemble amyloid fibrils and/or to inhibit their formation. In addition, natural polyphenolic compounds are considered as promising pharmaceuticals against amyloid diseases^10^. These features have also been observed in others aromatic compounds with structural similarity to polyphenols, such as hydroxyindoles^24^,^25^ or melatonin^26^.

Given the proven ability of polyphenols and indoles to disrupt amyloid fibril formation, a feature also observed in several pyridinium-type compounds^27^, we raised the question whether hydroxypyridines (HPs) could also act as inhibitors of fibrillization. Supporting this idea is the observation that pyridoxamine (PM), a natural derivative of vitamin B6, has a cytoprotective effect against the Aβ_1-42_ induced apoptosis^28^.

Here we have screened the effect of different natural (PM, PN and PL) and synthetic HPs (2HP, 4HP, 3HP, 5Cl-3HP, 2Cl-3HP, 2 m-3HP, 6 m-3HP and 2,6dm-3HP) (Fig. 1) on the fibrillogenesis of HEWL. Although most of the assayed HPs affected the fibrillization of HEWL, only *ortho-*methylated 3HPs (2m-3HP, 6m-3HP and 2,6dm-3HP) inhibited the HEWL fibril formation through direct binding onto the native monomeric HEWL structure.

## Results and Discussion

### Effect of different hydroxypyridines on the fibrillization propensity of HEWL

**Figure 1:**
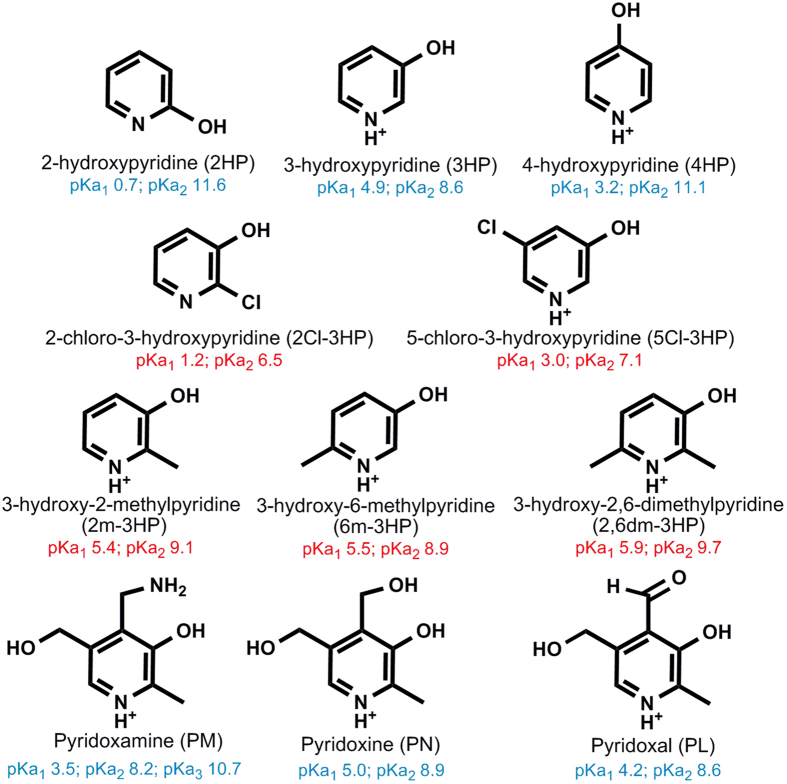
Chemical structures of the main ionic forms at pH 2.0 of the eleven different hydroxypyridines (HPs) used in this work. The protonation states of the pyridine and phenol groups were derived from the corresponding pKa values, which were either obtained from bibliography^59^,^60^ (*blue*) or determined spectrophotometrically in this work (*red*).

To study whether HPs can affect the fibrillization of HEWL we incubated different protein solutions in the absence or presence of HPs (Fig. 1) at a 1:100 molar ratio. Incubations were carried out during ~17d at pH 2.0 and 60 °C, conditions which are reported to induce amyloid fibril formation in HEWL^16^. ^1^H-NMR spectroscopy revealed that incubation of the different HPs under these experimental conditions did not result in any significant thermal degradation (only 2Cl-3HP and 2,6dm-3HP degraded ~10% after 17d of incubation). The kinetics of the fibril formation was monitored by the increase in the ThT fluorescence emission at 485 nm as a result of the ThT binding to amyloid β-strands^29^.

**Figure 2:**
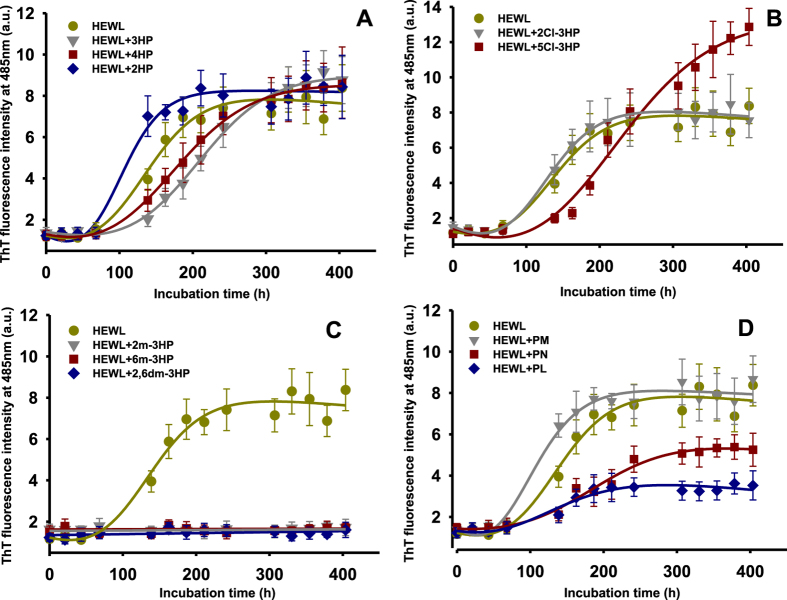
Effect of the different HPs at 100-fold molar excess on acid-induced HEWL fibrillogenesis as monitored by ThT fluorescence. For clarity of the representation, the data are spread in four different panels, each one of them gathering **(A)** the structurally simplest HPs, **(B)** the chlorinated 3HPs, **(C)** the *o*-methylated 3HPs, and **(C)** the natural vitamin B6 derivatives. Experimental data represent the average ± s.d. (*n* = *3*).

The time-dependent variation of the ThT signal when HEWL was incubated alone followed the typical profile of a nucleation-dependent pathway. The nucleation lag-phase was ~45 h after which the ThT fluorescence intensity increased rapidly until reaching a plateau (Fig. 2 and Table 1). This aggregation profile functioned as control to study if 2HP, 3HP or 4HP could interfere with the aggregation of HEWL. The presence of 2HP reduced the fibrillization lag-phase (~23%) while it increased the growth rate constant (*k*_*f*_) (Table 1). In contrast, the presence of 3HP and 4HP prolonged the fibrillization lag-phase and reduced the *k*_*f*_ value. Although 2HP, 3HP and 4HP affected the fibrillization kinetics in a different way, none of them changed the final amplitude of the aggregation curve, and therefore, inhibited the HEWL fibrillization (Fig. 2a and Table 1). These results show that different HPs interfere differently with the fibrillization of HEWL, and suggest that the position of the hydroxyl group on the pyridinic ring could somehow guide the mechanism through which the effect takes place.

Since 3HP was able to increase the duration of the aggregation lag-phase slightly more than 4HP, we focused the quest for potential inhibitors on different substituted 3HPs. First we studied the electron withdrawing effect using chlorine as substituent. The aggregation tendency of HEWL was practically unaltered in presence of 2Cl-3HP, while 5Cl-3HP increased the duration of the aggregation lag-phase, while at the same time significantly enhancing the amplitude of the aggregation curve (~100%) (Fig. 2b and Table 1). Thus, the chlorination of the 3HP ring reduced rather than improved its inhibitory capacity. Next, we analyzed the electron donating effect by using the methyl group as substituent. The time-dependent variation of the ThT signal when monomeric HEWL was incubated with 2 m-3HP, 6 m-3HP or 2,6dm-3HP was negligible (Fig. 2c), revealing that all these *o*-methylated 3HPs are potent inhibitors of HEWL fibrillization.

Likewise, natural derivatives of vitamin B6 are *o*-methylated 3HPs at C2. Given the inhibitory effect observed for 2 m-3HP, 6 m-3HP and 2,6dm-3HP, it would be expected that natural PM, PN and PL (see Fig. 1) could also inhibit HEWL fibril formation. However, the presence of PM did not change the *k*_*f*_ or the amplitude of the aggregation curve, although it reduced slightly the lag-phase (~15%). On the other hand, PN and PL reduced the *k*_*f*_ (~32 and 28% respectively) and the amplitude (~32 and 55% respectively) while increasing the lag-phase (~36 and 41% respectively) (Fig. 2d and Table 1). These results show that methylation of the 3HP ring might help to enhance its inhibitory effect, although it is not necessarily sufficient to completely inhibit the fibrillization of HEWL as additional ring substitutions (*e.g.* PM, PN and PL) can reduce the potency of *o*-methylated 3HPs. Therefore, other structural and/or electronic factors must be also involved in the inhibitory effect.

### Atomic force microscopy analysis of HEWL samples incubated in the presence of different hydroxypyridines

Although the ThT fluorescent results indicate that *o*-methylated 3HPs potently inhibit HEWL amyloid fibril formation, this does not rule out the possibility that other non-fibrillar assemblies could be formed in presence of *o*-methylated 3HPs. For instance, different spherical-like aggregates were previously detected as a collateral result of the inhibition of HEWL fibrillization by hydroxyindoles^25^ or glutathione^30^.

**Figure 3:**
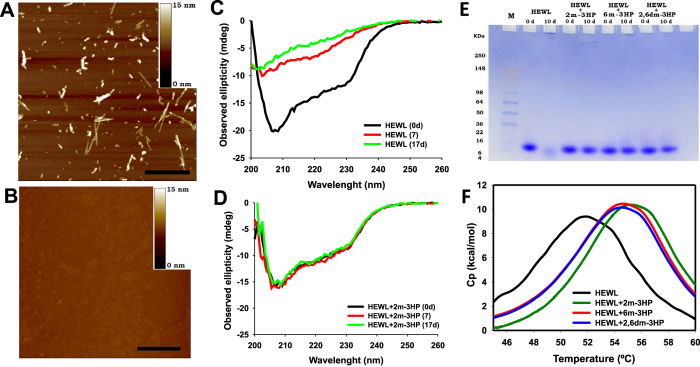
The effect of *o*-methylated 3HPs on: i) the HEWL fibril formation, ii) its α-to-β conformational rearrangement, iii) the HEWL acid-induced proteolysis, and iv) the HEWL thermal stability. **(A–B)** AFM micrographs of HEWL solutions (0.2 mM) previously incubated during 16d at pH 2.0 and 60 °C either **(A)** in buffer, or **(B)** in the presence of 20 mM 2 m-3HP. The scale bar represents 0.5 μm. **(C-D)** Far-UV CD spectra of HEWL incubated during 0, 7 and 17 days at pH 2.0 and 60 °C either **(C)** in buffer, or **(D)** in the presence of 20 mM 2 m-3HP. **(E)** SDS-PAGE gel analysis of solutions containing HEWL incubated at pH 2.0 and 60 °C during 0 and 10d either in the absence or in the presence of different *o*-methylated 3HPs. **(F)** Differential scanning calorimetry thermograms of different HEWL solutions (0.2 mM) at pH 2.0 acquired in the absence or in the presence of 20 mM of the different *o*-methylated 3HPs.

Hence, we used AFM to assess the existence of aggregates in the HEWL samples incubated in the presence of *o*-methylated 3HPs. The AFM micrograph of the HEWL sample incubated alone contained large quantities of unbranched fibrils of ~15nm in diameter and variable in length (Fig. 3a). In contrast, the AFM micrographs of the HEWL samples incubated with 2 m-3HP, 6 m-3HP and 2,6dm-3HP (Fig. 3b and Supplementary Fig. S1) did not show any fibrillar assemblies, nor the presence of other different morphological aggregates. Hence, their addition did not result in an increased population of toxic amyloid oligomers, as previously observed for the disassembly of fibrils from Aβ peptides^31^. The complete inhibition of the aggregation process mediated by *o*-methylated 3HPs is comparable with that of 3-hydroxyindole on Aβ_1-40_^24^ or (-)-epigallocatechin gallate on IAPP^32^. Our results indicate that *o*-methylated 3HPs are able to abolish the aggregation of HEWL at pH 2 and 60 °C. However, the addition of *o*-methylated 3HPs on preformed amyloid fibrils did not induce any disassembly event (Supplementary Fig. S2).

Additionally we used AFM to visualize if the other HPs could influence the HEWL amyloid fiber morphology. The fibers assembled in presence of the other HPs exhibited a similar morphology as those produced from HEWL incubated alone (Supplementary Figs S3–S5). However, as suggested by the ThT curves, the amount of fibers changed when using different HPs. The AFM micrograph of HEWL co-incubated with 2HP for 7d contained more fibrils than that from the sample including 4HP, while only a few fibrils were detected in the micrograph of the sample containing 3HP (Supplementary Fig. S3). The micrograph of HEWL co-incubated with 2Cl-3HP during 16d (Supplementary Fig. S4a) showed a number of fibrils comparable with the control (Fig. 3a), whereas the presence of 5Cl-3HP increased this amount substantially (Supplementary Fig. S4b). PL and PN reduced the number of fibrils formed after 16d of incubation, while the micrograph of the sample containing PM was similar of that obtained from HEWL incubated alone (Supplementary Fig. S5).

These results show that HPs completely inhibit (2 m-3HP, 6 m-3HP and 2,6dm-3HP) or delay (*e.g.* 3HP, PN, PL…) the amyloid fibril formation, but do not divert the HEWL aggregation mechanism towards different aggregation pathways.

### *O-*methylated 3-hydroxypyridines inhibit the conformational alteration of HEWL

Next we wondered what would be the molecular mechanism through which HPs, especially *o*-methylated 3HPs, can affect/inhibit the aggregation of HEWL. It is known that amyloid fibril assembly is driven by a structural rearrangement towards a β-sheet conformation^4^. Therefore, we used far-UV CD spectroscopy to investigate if HPs could influence the HEWL secondary structural conversion or, on the contrary, their action takes place at a different structural level.

The CD spectrum of HEWL obtained prior to incubation exhibited the typical profile of a predominantly α-helical conformation^17^. However, after 7d of incubation, the spectrum intensity decreased, probably resulting from the formation of insoluble aggregates (Fig. 3c) and the acid-induced hydrolysis (*see next section*). This variation, preserved after 17d, was concomitant with a change in the spectrum profile involving a reduction of the α-helicity (~78%) and a notably increase of the β-sheet content (Supplementary Table S1). A similar behavior was observed when HEWL was incubated with 2HP (Supplementary Fig. S6a), 2Cl-3HP, 5Cl-3HP (Supplementary Fig. S7), PM, PL or PN (Supplementary Fig. S8), proving that all these HPs cannot prevent the conformational alteration accompanying the amyloid assembly.

Neither 3HP nor 4HP prevented the structural transition of HEWL towards a highly amyloidogenic β-sheet rich conformation (Supplementary Table S1). However, their presence reduced the CD spectrum intensity decrease as observed when HEWL was incubated alone, thus suggesting that 3HP and 4HP are enhancing the solubility of the β-sheet rich conformations (Supplementary Fig. S6b,c).

On the contrary, the CD spectrum profile of HEWL remained unaltered after 17d of co-incubation with 2 m-3HP, 6 m-3HP or 2,6dm-3HP (Fig. 3d, Supplementary Fig. S9 and Table S1), indicating that these compounds are able to completely inhibit the α-to-β conformational transition.

### *O-*methylated 3-hydroxypyridines inhibit acidic proteolysis

Our data reveal that *o*-methylated 3HPs can inhibit the aggregation of HEWL through blocking the α-to-β conformational rearrangement. We then wondered whether this blockage would also affect the acidic proteolysis of HEWL, which was linked to its acid-induced fibrillization process. Several reports concluded that HEWL undergoes partial fragmentation (at Asp-X residues) under acidic conditions producing the amyloidogenic fragments G49–D101 and Y53–101D^33^. Therefore, we used SDS-PAGE to study if HPs had any protective role on the HEWL acid hydrolysis.

Aliquots of the reaction mixtures were withdrawn at 0 and 10d of incubation and analyzed on SDS-PAGE. The HEWL sample incubated alone displayed high molecular weight assemblies in the well of the stacking gel, but also a significant population of bands corresponding to molecular weights lower than 14.3 kD that can be attributed to the acid-induced hydrolytic polypeptide fragments (Fig. 3e and Supplementary Fig. S10) as evidenced MALDI-TOF/TOF analysis (Supplementary Fig. S11). A similar pattern was observed on the gels for HEWL incubated with 2HP, 4HP, 2Cl–3HP, 5Cl–3HP, PN, PL or PM (Supplementary Fig. S10), proving that these compounds do not protect against the acidic proteolysis. The presence of 3HP reduced the acidic proteolysis, although high molecular assemblies were still visible (Supplementary Fig. S10a). However, these high molecular weight assemblies were not detected in the aliquots of HEWL incubated with 2 m-3HP, 6 m-3HP or 2,6dm-3HP. After 10d of incubation, only the band corresponding to native HEWL (14.3 kD) appeared in the gel, lacking other bands attributable to lower molecular weight peptides (Fig. 3e).

This SDS-PAGE analysis confirms that *o*-methylated 3HPs inhibit the formation of high molecular weight assemblies, but also the acidic proteolysis and consequent formation of potential amyloidogenic peptides.

### Effect of *o-*methylated 3-hydroxypyridines on the thermal stability of HEWL

The fibrillization propensity of HEWL not only depends on the hydrophobicity of core residues^34^, but it is strongly correlated with its thermal stability, which is pH-dependent^35^. Small molecules acting as inhibitors of HEWL aggregation are known to increase HEWL thermal stability through a direct binding process^36^. Hence, if *o*-methylated 3HPs can modify the thermal stability of HEWL it would suggest that the inhibition of HEWL fibrillization could occur through direct binding.

Hence, we performed differential scanning calorimetry experiments on HEWL solutions prepared in the absence and in the presence of 2 m-3HP, 6 m-3HP and 2,6dm-3HP. All the calorimetric curves exhibited a unique heat absorption peak, characteristic of the two-state reversible thermal denaturation transition described for HEWL^37^. However, the addition of *o*-methylated 3HPs clearly enhanced the heat capacity and the enthalpy of unfolding, while increasing the *T*_m_ by ~2.7 °C when 6 m- or 2,6dm-3HPs were added, and by ~3.6 °C in the presence of 2 m-3HP (Fig. 3f). The presence of other HPs did not especially influence the thermal denaturation of HEWL (Supplementary Fig. S12).

These results reveal that *o*-methylated 3HPs increase the thermal stability of HEWL, and point towards a direct binding process occurring between these 3HPs and the monomeric HEWL.

### *O-*methylated 3-hydroxypyridines interact with monomeric HEWL at two distinct binding sites

**Figure 4:**
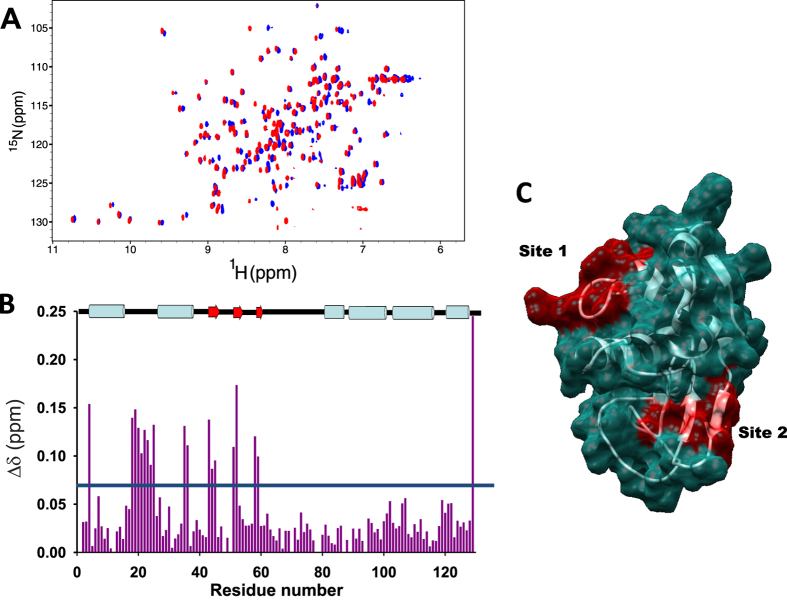
Nuclear magnetic resonance study of the solution interaction between HEWL and 2 m-3HP. **(A)** Overlapping of the overall ^15^N-HSQC spectra of HEWL at pH 2.0 and 37 °C acquired in the absence (*red*) or in the presence of 2 m-3HP (*blue*) at a HEWL:2 m-3HP ratio of 1:5. **(B)** Chemical shift histogram of the weighted amide chemical shift perturbation observed along the HEWL sequence after addition of 2 m-3HP at a HEWL:2 m-3HP ratio of 1:5. The secondary structure is indicated on the plot as reference. **(C)** Cartoon representation of the HEWL structure and its surface generated using PyMOL and the crystal structure with the PDB code 1LSE. Amino acids whose resonances were affected by the presence of 2m-3HP (above the blue line in panel B; Δδ_ave_ > 0.07 ppm) are coloured in red.

We used NMR spectroscopy to map the interaction region of the different *o*-methylated 3HPs onto the monomeric HEWL. The ^15^N-HSQC spectrum of HEWL was clearly affected upon increasing the 2 m-3HP (Fig. 4a), 6 m-3HP or 2,6dm-3HP concentrations, which confirm the direct binding between these 3HPs and monomeric HEWL. Upon 2 m-3HP addition, the ^15^N-HSQC spectrum revealed detectable chemical shift perturbations (Fig. 4b) that were similar to those occurring with 6 m-3HP or 2,6dm-3HP (Supplementary Fig. S13). The largest variations (Δδ > 0.08 ppm) involved the resonances of G4, D18–L25, E35–S36, T43–N46, T51–D52, I58–N59 and L129. These were mapped onto the structure of HEWL revealing that *o*-methylated 3HPs bind to two different regions (Fig. 4c). One binding site mainly involves the unstructured region located between the helices A and B in the α-domain (*site 1* in Fig. 4c), while the second binding site is located at the β-domain (*site 2* in Fig. 4c). This region cooperatively unfolds with the C-helix constituting the critical event that triggers the aggregation of human lysozyme^38^. Although chemical shift perturbations follow the typical profile of a binding event, we cannot rule out that they could hold certain contributions of long-range interactions due to intramolecular communication pathways.

We then used fluorescent quenching to evaluate the corresponding association constants (*K*_*a*_ and *K*_*b*_), as has already been done when studying other HEWL-ligand interactions^39^. Most of the HEWL fluorescence (>80%) arises from W62 and W108^40^, therefore the binding of UV-active *o*-methylated 3HPs relatively close to these residues would be expected to perturb the intrinsic HEWL fluorescence.

The intensity of the fluorescent spectrum of HEWL decreased with increasing concentrations of 2 m-3HP, 6 m-3HP or 2,6dm-3HP, through a mechanism that likely involves a combined dynamic and static quenching process, as evidenced by the upward curvature in the Stern-Volmer plot^41^ (Supplementary Fig. S14). All the titration plots displayed exponential decay profiles, which suggest the lack of cooperative events occurring between the two binding sites. The thermodynamic binding data were fitted to a two-binding site mechanism, which gave lower residuals than when using a single binding site scheme (Supplementary Fig. S15). The obtained association constants demonstrated that the different *o*-methylated 3HPs are able to bind HEWL with strong affinity (*K*_*a*_ > 7·10^3^ M^−1^; *K*_*b*_ > 4·10^2^ M^−1^). *K*_*a*_ was always higher than *K*_*b*_ indicating that the two binding sites have different affinities towards the *o*-methylated 3HPs. In the presence of 2m-3HP or 6m-3HP, *K*_*a*_ was six times higher than *K*_*b*_, whereas *K*_*a*_ ≈ 22·*K*_*b*_ in the case of 2,6dm-3HP. Among the different *o-*methylated 3HPs, 2m-3HP was the compound with the higher affinity for HEWL (Table 2).

The results prove that different *o*-methylated 3HPs can bind independently to monomeric HEWL at two distinct binding sites with different affinity.

### Mapping the interactions between *o-*methylated 3HPs and HEWL by molecular docking simulations

**Figure 5:**
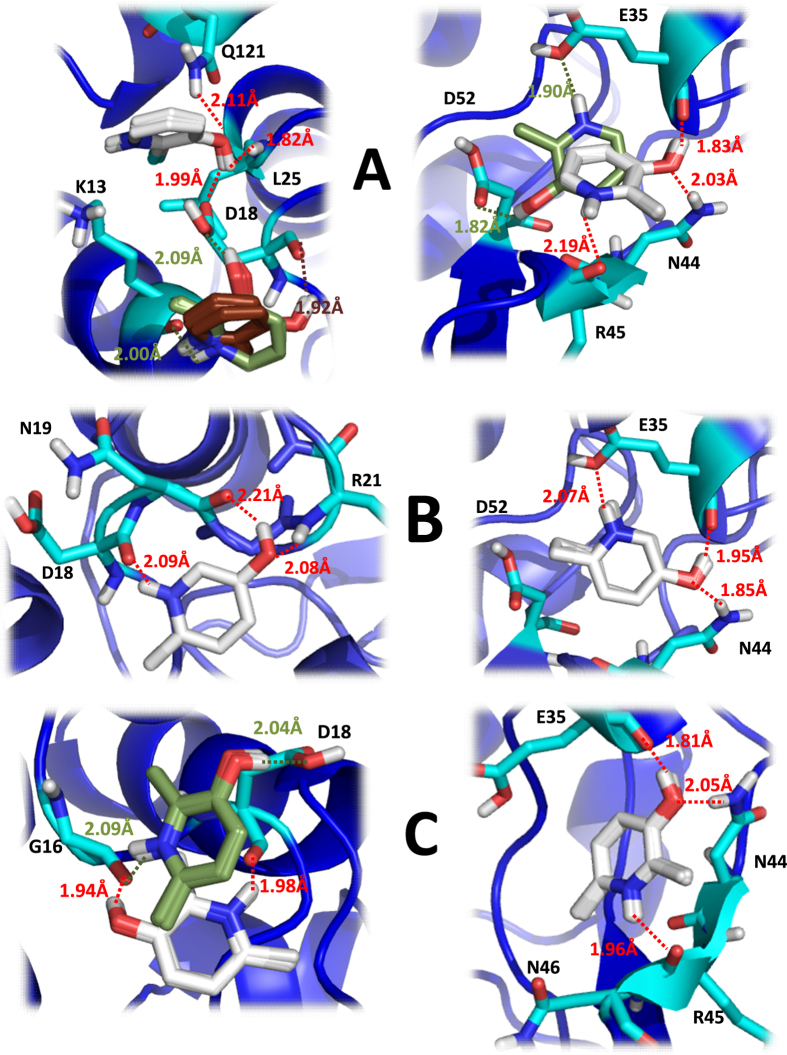
The docking pose/s of (A) 2 m-3HP, (B) 6 m-3HP and (C) 2,6dm-3HP generated for the HEWL binding sites 1 (*left*) and 2 (*right*). The different ligand clusters found for each binding site are colored as white, brown or green, a color code that reflects the Autodock energy from the lowest to the higher energy, respectively. When all twenty ligand structures were jointly clustered, the ligands are merely shown in white. In all panels the interacting residues are shown as atom-coloured sticks, while the HEWL structure (previously equilibrated by MD simulations during 100 ns at 27 °C) is shown as blue ribbons. Images were generated using PyMOL software.

We then used molecular docking simulations to gain deeper atomic insights on the intermolecular interactions occurring between *o*-methylated 3HPs and HEWL. Calculations performed on binding *site 1* (Fig. 4c) revealed that the three cationic *o*-methylated 3HPs interact differently on this site. When 2 m-3HP was used as ligand, the twenty models were clustered into three different poses (Fig. 5a*;left*) exhibiting similar binding energy (Supplementary Table S2). In one of them, 2 m-3HP was linked to the protein via the formation of three different H-bonds between its hydroxyl group and the amide proton of L25 and the side chains of D18 and Q121. In the two remaining poses, the ligand exhibited two donating H-bonds through its hydroxyl and pyridinic protons with the amide carbonyl of K13 and the side chain or the amide carbonyl of D18. On the contrary, 6 m-3HP displayed all twenty models were clustered into a single pose, which was linked to the protein forming three different H-bonds between its hydroxyl/pyridine groups and the backbone donating/accepting groups of D18, N19 and R21 (Fig. 5b;*left*). 2,6dm-3HP showed two different clusters linked to *site 1*, establishing both of them two donating H-bonds between their hydroxyl and pyridinic protons with the amide carbonyl of G16 and the side chain or the amide carbonyl of D18 (Fig. 5c;*left*).

These results suggest that the different *o*-methylated 3HPs do not have a common preferable interaction pattern for the binding *site 1*. Hence, it seems that the chemical structure of each ligand directs its most energy favored interaction. Moreover, the conformational flexibility of the disordered region between the helices A and B (H15-G26), as well as the high population of solvent exposed donating/accepting H-bonds groups (Supplementary Fig. S16) might facilitate the accommodation of *o*-methylated 3HPs within the binding region. Therefore, we argue that these compounds are able to explore different positions on the binding *site 1*.

Calculations carried out on *site 2* (Fig. 4c) revealed that the binding profile is conserved upon interaction with different *o*-methylated 3HPs, which involved the participation of E35, N44, R45 and D52 (Supplementary Table S2). Two equally populated clusters were observed in a fairly close position (RMS ~2 Å) when 2m-3HP was modelled whereby the ligand displayed either two donating H-bonds with the side chains of E35 and D52, or three H-bonds with the amide carbonyls of E35 and R45 and the side chain of N44 (Fig. 5a;*right*). The twenty docked models were clustered in a unique pose when 6 m-3HP and 2,6dm-3HP were used as ligands. Both ligands showed a donating H-bond between their hydroxyl proton and the amide carbonyl of E35, and an accepting H-bond between their hydroxyl oxygen and the amide proton of N44 side chain. The third donating H-bond was different between them. While for 6 m-3HP the H-bond was established between the pyridinic proton and the side chain of E35, in the case of 2,6dm-3HP the most energy favored H-bond was formed with the amide carbonyl of R45 (Fig. 5b,c;*right*).

The docking results obtained for the binding *site 2* suggest that *o*-methylated 3HPs exhibit a higher residue specificity than that displayed in *site 1*. Moreover, it seems that the different methyl substitution somehow directs the most favored orientation of the ligand within the *site 2*. However, none of the binding poses exhibit an interaction involving the methyl group, which suggest that this substituent is not directly involved in the binding process.

Autodock binding energy has been used to determine qualitatively the most favored binding sites or conformations in many ligand-protein interactions^42^. The averaged docking binding energy is consistently higher on binding *site 2* than that on *site 1* (ΔE ~ 0.8–1.0 kcal/mol) (Supplementary Table S2), which points to a lower affinity and specificity of *site 1* than *site 2*. Moreover, these results suggest that *K*_*a*_ could be ascribed to the interaction between *o*-methylated 3HPs and *site 2*, while *K*_*b*_ would embody the binding constant with *site 1* (Table 2).

### *O-*methylated 3-hydroxypyridines do not bind pre-existing HEWL amyloid fibrils

NMR measurements and docking studies have proved that *o*-methylated 3HPs bind monomeric HEWL. However, we do not have any insight yet whether *o*-methylated 3HPs could also bind HEWL fibrils and thus, additionally act on different aggregation pathways.

Hence we acquired the ^1^H-NMR spectra of the three *o*-methylated 3HPs in the absence and in the presence of pre-formed HEWL fibrils. The ^1^H-NMR signals corresponding to the methyl/s and the aromatic protons still appeared in the presence of fibrils, which proves that these compounds do not bind on pre-existing fibrils. This was not the case when using ThT as a positive control (ThT binds amyloid β-strands^29^), since its ^1^H-NMR signals disappeared as a result of the long molecular tumbling time of the ThT-fibril complex (Supplementary Fig. S17).

This NMR data prove that after the misfolding took place, *o*-methylated 3HPs are no longer able to bind HEWL. Consequently, these compounds are only able to acting as inhibitors of the forward aggregation process.

### All observations in perspective: rationale for the inhibition of HEWL fibrillization by *o*-methylated 3-hydroxypyridines

HEWL is a polycationic protein at acidic pH (pI~11.3)^43^. Therefore, at low pH the electrostatic repulsion should be dominant over hydrogen bonding or hydrophobic driving forces. Hence, electrostatic repulsion should act as a barrier to the self-assembly of HEWL. Likewise, it seems contradictory that cationic *o*-methylated 3HPs (predominantly at pH 2.0; Fig. 1) could bind polycationic HEWL and inhibit its fibrillization. However aggregation of HEWL is strongly modulated by salts, which act as counter-ions to the positive charged HEWL^44^ making van der Waals attractions gain relevance over electrostatic repulsions. The fibrillization lag-phase is shortened upon increasing salt concentrations, but this occurs only up to ~350 mM, since at higher concentrations amorphous precipitation sets in^45^. Hence, Hill *et al.* suggested that repulsive charge interactions are prerequisite for HEWL fibril assembly^45^. These results were also theoretically confirmed by G. Pellicane, who used the DLVO theory to demonstrate that HEWL fibrillization is guided by an essential competition between short range attractions and long range repulsions^46^.

Positive charge interactions are not only crucial for HEWL fibrillation. At residue level, arginine-arginine contact pairs have been detected in several proteins^47^. In addition, cationic groups in di-histidine and other histidine-based peptides exhibit attractive interactions regardless the strong Coulomb repulsion forces^48^.

Water solvation, the effect of counter-ions, and the possibility of establishing cationic contact pairs probably constitute the driving forces that make the interactions between polycationic HEWL and cationic HPs energetically favorable. Moreover, the interaction of cationic *o*-methylated 3HPs on *site 2* can be additionally favored by the reduced electrostatic potential of this site, even at pH 2.0 (Supplementary Fig. S18).

**Figure 6:**
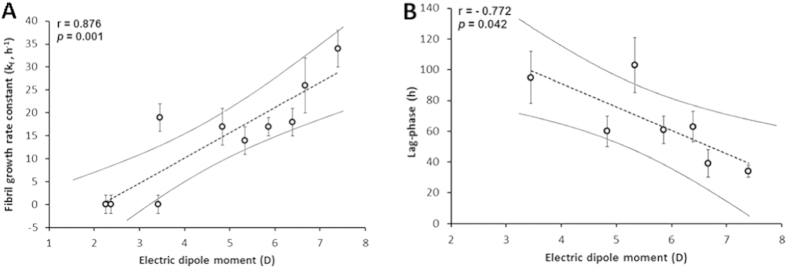
Linear regression between the electric dipole moment and (A) the fibril growth rate constant or (B) the fibrillization lag-phase. The dotted lines represent the best fit. The solid lines are its 95% of confidence bands. Values are expressed as average ± standard error. As HEWL did not aggregate in the presence of 2 m-, 6 m- and 2,6dm-3HP, the corresponding data points were excluded from plot B (Fig. 2c, Table 1).

However, this does not explain how *o*-methylated 3HPs have a higher capacity to inhibit HEWL fibrillization than other HPs. Trying to shed light on this aspect, first we determined the pKa values of those HPs that were not found in literature (i.e. 2Cl-3HP, 5Cl-3HP, 2 m-3HP, 6 m-3HP and 2,6dm-3HP)(Supplementary Fig. S19). The pKa values of most HPs were well above 2, expect those corresponding to 2HP and 2Cl-3HP (Fig. 1). The main ionic forms at pH 2 were geometrically optimized using DFT calculations. While the computed atomic charges were similar between the different HPs (Supplementary Tables S3–S5), the electric dipole moment (EDM) notably diverged between them (Supplementary Table S6). From this observation, we then studied whether the EDM could somehow correlate with the experimentally determined indicators of fibrillization (i.e. *k*_*f*_ or the lag-phase (Table 1)). The obtained plots revealed a strong positive correlation of the EDM with the *k*_*f*_ (r > +0.88; *p* < 0.05), as well as a strong negative correlation between the EDM and the fibrillation lag-phase (r < −0.77; *p* < 0.05) (Fig. 6).

All the studied HPs are cationic heteroaromatic compounds possessing two groups capable to accept or/and donate hydrogen bonds. However, only cationic 2 m-3HP, 6 m-3HP and 2,6dm-3HP have an unusual reduced EDM. Therefore, it is tempting to speculate that this lower EDM could facilitate their ability to interact with HEWL, constraining its structure and therefore, inhibiting the amyloid fibril formation.

## Methods

### Amyloid fibril formation of HEWL

Solutions of HEWL (0.2 mM) were prepared in the absence or in the presence of the different HPs (20 mM) by using an aqueous solution containing 137 mM NaCl and 2.6 mM KCl at pH 2.0. The resulting mixtures were first vortexed and then incubated at 60 °C to induce HEWL fibrillization. Aliquots at different incubation times were taken for analysis.

### Thioflavin T (ThT) Fluorescence Assay

Aliquots corresponding to the different HEWL/HPs reaction mixtures were taken at different incubation times and diluted in milli-Q water to a final HEWL concentration of 4 μM. The samples were then mixed thoroughly with 4 μl of a ThT stock solution (5 mM) to a final ThT concentration of 20 μM, prior to measuring the ThT fluorescence emission intensities at 485 nm (λ_exc_ 440 nm). Fluorescent measurements were carried out at 37 °C on a Cary Eclipse fluorescence spectrophotometer equipped with a Peltier temperature controlled cell holder. The data from ThT fluorescence measurements were fit to the following sigmoidal equation^49^ using SigmaPlot:


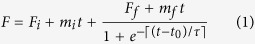


where *F* is the time-dependent fluorescent intensity and *t*_*0*_ is the time to reach 50% of maximal fluorescence. The initial base line during the lag time is described by *F*_*i*_ + *m*_*i*_*t*. The final base line after the growth phase has ended is described by *F*_*f*_ +  *m*_*f*_*t*. The apparent rate constant (*k*_*f*_) for the growth of fibrils is given by 1/τ, the lag time is calculated as *t*_0_ − 2τ, and the amplitude is given by *F*_*f*_ − *F*_*i*_.

### Atomic force microscopy (AFM)

Aliquots (10 μl) from each HEWL/HP reaction mixture were collected after different incubation times and diluted in 90 μl of milli-Q water. A volume of 50 μl of the resulting dilutions were placed onto the mica surface and incubated for 5 min at room temperature before drying with N_2_ gas. The mica was rinsed 5 times with 1ml milli-Q water and dried with N_2_ gas before observation under a Veeco Multimode atomic force microscope equipped with a NanoScope IV controller. The particle widths were measured using the NanoScope SPM v5 software taking into account the tip-sample dilation effects that were modeled by assuming the tip to be a hemisphere with a radius larger than the radius of the measured specimen. The width (*w*) of the measured fibril was calculated from the following relation between the tip radius (*r*) and the apparent particle width (*w*_a_): *w* = 2(*w*_a_^2^/16*r*)^50^.

### SDS-PAGE electrophoresis

Aliquots (4 μl) corresponding to the different HEWL/HP reaction mixtures were taken at 0 and 10d of incubation and mixed with 4 μl of Laemmli sample buffer (Bio-Rad) containing 50mM DTT. The resulting samples were then subjected to SDS-PAGE analysis by using 4–20% Mini-Protean© TGX precast gels (Bio-Rad). Proteins were visualized with Coomassie blue R-250 (Bio-Rad).

### Circular dichroism (CD) analysis

Aliquots from each HEWL/HP reaction mixture were collected after 0, 5.7, 8.7 and 15.7d of incubation at 60 °C and diluted in the same reaction medium to a final HEWL concentration of 50 μM. Samples were then subjected to CD analysis using a J-715 spectropolarimeter (JASCO). Far-UV CD spectra were recorded between 260–200 nm in a quartz cell of 0.2 mm path length. The scan speed was 50 nm/min with a response time of 1s and a step resolution of 0.2 nm, while 5 scans were accumulated. Base-line spectrum was subtracted for all spectra. The secondary structure content was derived from the corresponding far-UV CD spectrum by using the BeStSel on-line platform (http://bestsel.elte.hu/).

### Differential scanning calorimetry (DSC)

Solutions containing HEWL (50 μM) alone or in presence of 2.5 mM of 2-HP, PM, 2Cl-3HP, 2 m-3HP, 6 m-3HP or 2,6dm-3HP were prepared in a 20 mM chloroacetate buffer at pH 2.0. The resulting solutions were first filtered through a 0.22 μm membrane and then degassed under vacuum for 10 min. The samples were placed in a Nano-DSC (TA instruments) equipped with capillary cells, and kept at 25 °C during 15 min to achieve the thermal equilibration of the cell. The reference cell was filled with 300 μl of degassed chloroacetate buffer (pH 2.0) and the sample cell with 300 μl of protein sample. Samples were scanned at 1 °C/min from 25 to 95 °C at 3atm pressure. Unfolding transition temperatures and enthalpies of the denaturation process were obtained by fitting the experimental data to the two-state model^37^, verified for HEWL, by using the NanoAnalyze (v.2.3.6) software.

### NMR spectroscopy

Interactions between 2 m-3HP, 6 m-3HP or 2,6dm-3HP and HEWL were mapped at residue level using NMR spectroscopy. 2D ^15^N-HSQC spectra were recorded at natural abundance for 5 mM HEWL solutions prepared in 20 mM sodium chloroacetate at pH 2.0 (90% H_2_O/10% D_2_O) in the presence of 0, 5, 12.5 and 25 mM HPs. Amide chemical shift perturbation was quantified as the average chemical shift change of the amide protons and nitrogens:


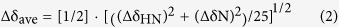


The chemical shift assignment for the amide protons and nitrogens of HEWL at pH 2.0 was transferred from that previously published at pH 3.5 and 37 °C^51^ using the ^15^N-HSQC spectra of HEWL acquired at pH 3.5, 3.0, 2.5, and 2.0. Proton chemical shifts were referenced to the water signal at 37 °C. ^15^N chemical shifts were referenced indirectly using the ^1^H,X frequency ratios of the zero-point. All the spectra were processed using NMRPipe/NMRDraw^52^, analyzed by Xeasy/Cara^53^ and plotted using Sparky software. UCSF Chimera was used for structural representations.

Aqueous solutions containing the different HPs (5 mM), 137 mM NaCl, 2.6 mM KCl and 1.6 mM DSS (added as internal reference) at pH 2.0 were incubated at 60 °C during 17d. 1D ^1^H-NMR spectra were collected for each sample before and after incubation. To determine the thermal stability of each HP after 17d of incubation, aromatic signals were integrated before and after incubation. Signals were internally referenced to the integral value of the DSS methyl signal.

NMR spectroscopy was also used to assess the interaction between *o*-methylated 3HPs and HEWL pre-existing amyloid fibrils. 1D ^1^H-NMR spectra of solutions containing either 0.25 mM of ThT (used as a positive control), 2 m-3HP, 6 m-3HP or 2,6 dm-3HP in presence of 137 mM NaCl and 2.6 mM KCl at pH 2.0 were acquired before and after the addition of preformed HEWL amyloid fibrils to a protein concentration of 0.5 mM.

All the NMR spectra were acquired at 37 °C on a Bruker Avance III operating at 600 MHz and equipped with a 5-m ^13^C,^15^N,^1^H triple resonance cryoprobe.

### Binding constant measurement

A 3ml solution containing HEWL (10 μM) in 2 mM sodium chloroacetate buffer at pH 2.0 was titrated by successive additions of a 2.5 mM stock solution of 2 m-3HP, 6 m-3HP or 2,6dm-3HP prepared in the same buffer. Titrations were done into a quartz cuvette until the concentration of *o*-methylated 3HP was 526 μM. Fluorescent spectrum at each titration point was measured at 37 °C on a Cary Eclipse fluorescence spectrophotometer equipped with a Peltier controller cell holder between 300 and 450 nm (λ_exc_ 280 nm). Fluorescent intensity data (*I*_F_) collected at 340nm was corrected for the inner filter effects caused by absorption of both exciting and emitted radiation by applying the following equation as reported by Lakowicz^54^:


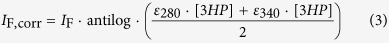


The corrected fluorescent intensity data (*I*_F,corr_) collected at 340 nm were then fitted to a model involving one or two independent binding sites using Dynafit software^55^. The multivariable models used during the fitting process considered the simultaneous variation of the *o*-methylated 3HPs and the HEWL concentrations at each titration point (Supplementary Fig. S20).

### Molecular docking of 2 m-3HP, 6 m-3HP and 2,6dm-3HP to HEWL

Molecular docking simulations were carried out to understand the binding architecture of 2 m-3HP, 6 m-3HP and 2,6dm-3HP (acting as ligands) on the crystal structure of HEWL. Initially, the structures corresponding to the cationic ligands (predominant species at pH 2.0; Fig. 1) were built using GaussView 5.0 and geometry optimized at the B3LYP/6–311++G(d,p) level of theory using the Gaussian 09 software^56^. The HEWL structure employed for docking studies was 1LSE.pdb with minor structural modifications to better simulate our experimental conditions (pH 2.0). The carboxylate at the C-terminus and acidic side chains were protonated, except those corresponding to D48 and D66 because of their low pKa (<1.5)^57^. Afterwards, the resulting structure was equilibrated at 27 °C during 100 ns using MD simulations. For the docking study, two grid volumes were built centered at the biding sites 1 (41 × 41 × 47 points; 0.375 Å grip spacing) and 2 (47 × 43 × 47 points; 0.375 Å grip spacing), previously mapped by NMR. Twenty binding models for each binding site were generated, each of them picked from an individual run as the lowest energy conformation among a maximum of 2,500,000 evaluations. The protein model was kept rigid during the docking, while torsions were allowed only for the ligands. Automated docking studies were performed using Autodock 4.1^58^ and the Lamarckian Genetic Algorithm as searching procedure.

## Additional Information

**How to cite this article**: Mariño, L. *et al.* Ortho-methylated 3-hydroxypyridines hinder hen egg-white lysozyme fibrillogenesis. *Sci. Rep.* **5**, 12052; doi: 10.1038/srep12052 (2015).

## Supplementary Material

Supplementary Information

## Figures and Tables

**Table 1:** Kinetic parameters of HEWL fibrillization in the presence of different HPs as determined by ThT binding assays.

	Growth rate constant *k*_*f*_^a^ (h^−1^)·10^#x02212;3^	Lag time^a^ (h)	Amplitude^a^ (a.u.)
HEWL	25 ± 4	45 ± 7	7.1
HEWL + 2HP	34 ± 4	34 ± 4	6.8
HEWL + 3HP	19 ± 3	96 ± 17	7.3
HEWL + 4HP	17 ± 4	60 ± 10	7.7
HEWL + 2Cl-3HP	26 ± 6	39 ± 9	6.8
HEWL + 5Cl-3HP	14 ± 3	103 ± 18	14.3
HEWL + 2 m-3HP	~0^b^	—c	0.1
HEWL + 6 m-3HP	~0^b^	—c	0.05
HEWL + 2,6dm-3HP	~0^b^	—c	0.4
HEWL + PM	28 ± 5	38 ± 9	7.3
HEWL + PN	17 ± 2	61 ± 9	4.85
HEWL + PL	18 ± 3	63 ± 10	3.21

^a^Kinetic parameters, *k*_*f*_, lag time, and the amplitude, were determined by fitting ThT fluorescence intensity *versus* incubation time (Fig. 2) to Equation 1. Errors show the standard errors of the average.

^b^The growth rate constant was considered as negligible for the reaction mixtures including 2 m-, 6 m- and 2,6dm-3HPs since the temporal increase in the ThT signal was lacking.

^c^The lag-time could not be determined in the presence of 2 m-, 6 m- and 2,6dm-3HPs since aggregation was not observed.

**Table 2:** Equilibrium constants between the different *o*-methylated 3HPs and each one of the two binding sites observed for HEWL as a result of its NMR titration using the different *o*-methylated 3HPs as titrants.

	*K*_*a*_ (M^−1^)·10^−3(^a)	*K*_*b*_ (M^−1^)·10^−3(^a)
HEWL + 2 m-3HP	9.3 ± 0.2	1.5 ± 0.2
HEWL + 6 m-3HP	7.4 ± 0.1	1.3 ± 0.1
HEWL + 2,6dm-3HP	8.9 ± 0.2	0.46 ± 0.13

^a^Equilibrium constants were determined by fitting the thermodynamic fluorescent data (340 nm) to a two-binding site mechanism using the DynaFit software^55^.
