# Supplementary material for: Ortho-methylated 3-hydroxypyridines hinder hen egg-white lysozyme fibrillogenesis
Source: Sci Rep. 2015 Jul 14;5:12052. doi: 10.1038/srep12052 (PMC4500996; doi:10.1038/srep12052)
Supplement: Supplementary Information [file srep12052-s1.pdf]

Supplementary Information for

Ortho-methylated 3-hydroxypyridines hinder hen egg-white lysozyme fibrillogenesis

Laura Mariño[1], Kris Pauwels[2,3], Rodrigo Casasnovas[1], Pilar Sanchís[1], Bartolomé Vilanova[1], Francisco Muñoz[1], Josefa Donoso[1] and Miquel Adrover[1*]

[1]Institut Universitari d'Investigació en Ciències de la Salut (IUNICS). Departament de Química, Universitat de les Illes Balears, Ctra. Valldemossa km 7.5, E-07122 Palma de Mallorca, Spain.

[2]Structural Biology Brussels, Vrije Universiteit Brussel, Pleinlaan 2, 1050 Brussel, Belgium.

[3]VIB Structural Biology Research Centre, Vlaams Instituut voor Biotechnologie, Pleinlaan 2, 1050 Brussel, Belgium.

*Correspondence to: Miquel Adrover, University of Balearic Islands, Phone: +34 971 173491; Fax +34 971 173426; e-mail: miquel.adrover@uib.es

Supporting Figures

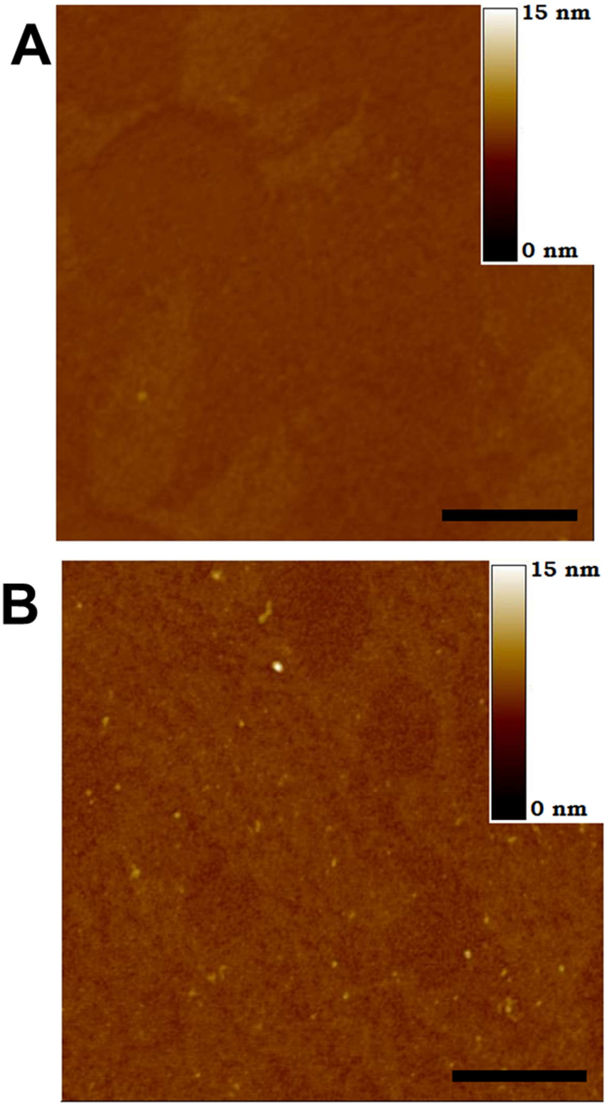

Supplementary Figure S1. AFM micrographs of HEWL solutions (0.2mM) previously incubated during 16d at pH 2.0 and 60°C in the presence of 20mM of **(A)** 6m-3HP or **(B)** 2,6dm-3HP. The scale bar represents 0.5μm.

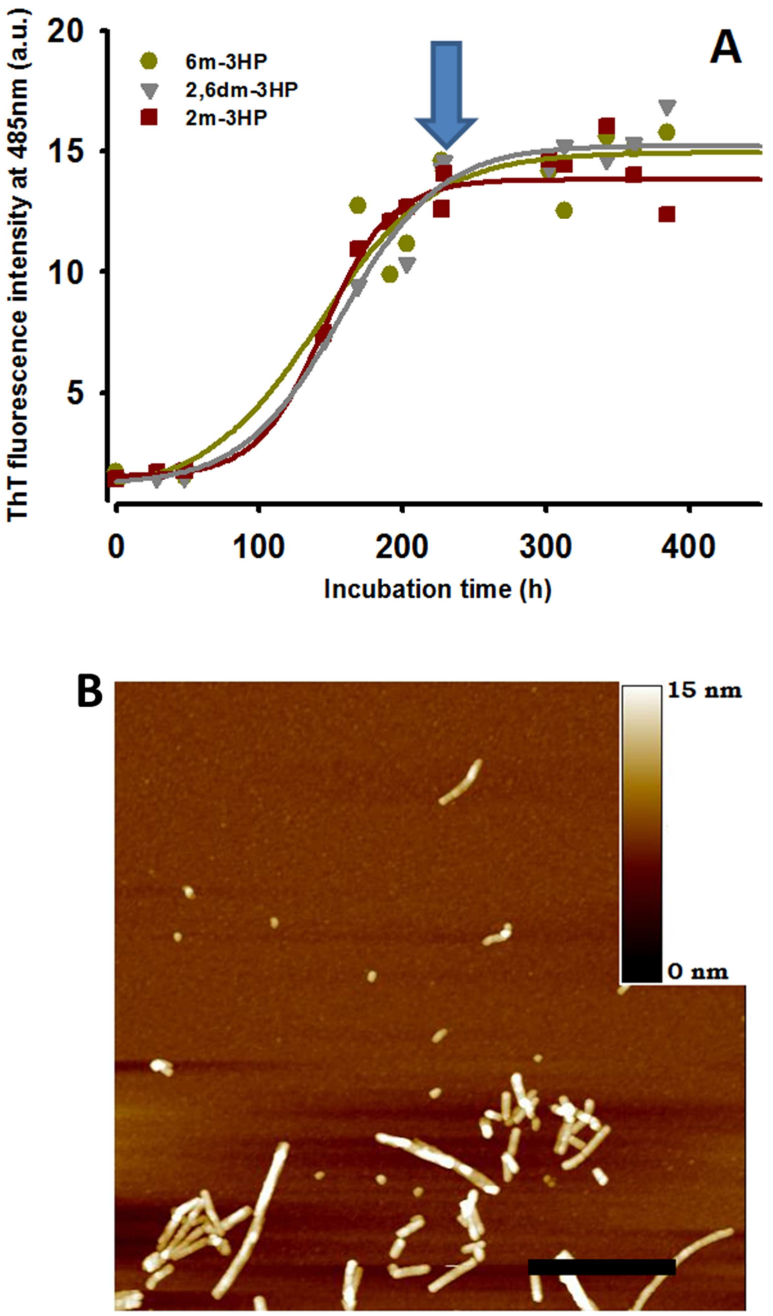

Supplementary Figure S2. Effect of different *o*-methylated 3HPs on preexisting HEWL amyloid fibrils. **(A)** ThT fluorescence intensity of different HEWL solutions (0.2mM) incubated at pH 2.0 and 60°C during 16d. The blue arrow represents the time-point (~10d) where different *o*-methylated 3HPs (20mM) were added to the incubated solution. **(B)** AFM micrograph obtained at 16d of incubation of a HEWL solution (0.2mM) that was previously incubated during 10d alone (pH 2.0 and 60°C) and, from day 10 to day 16 in presence of 20mM of 2m-3HP.

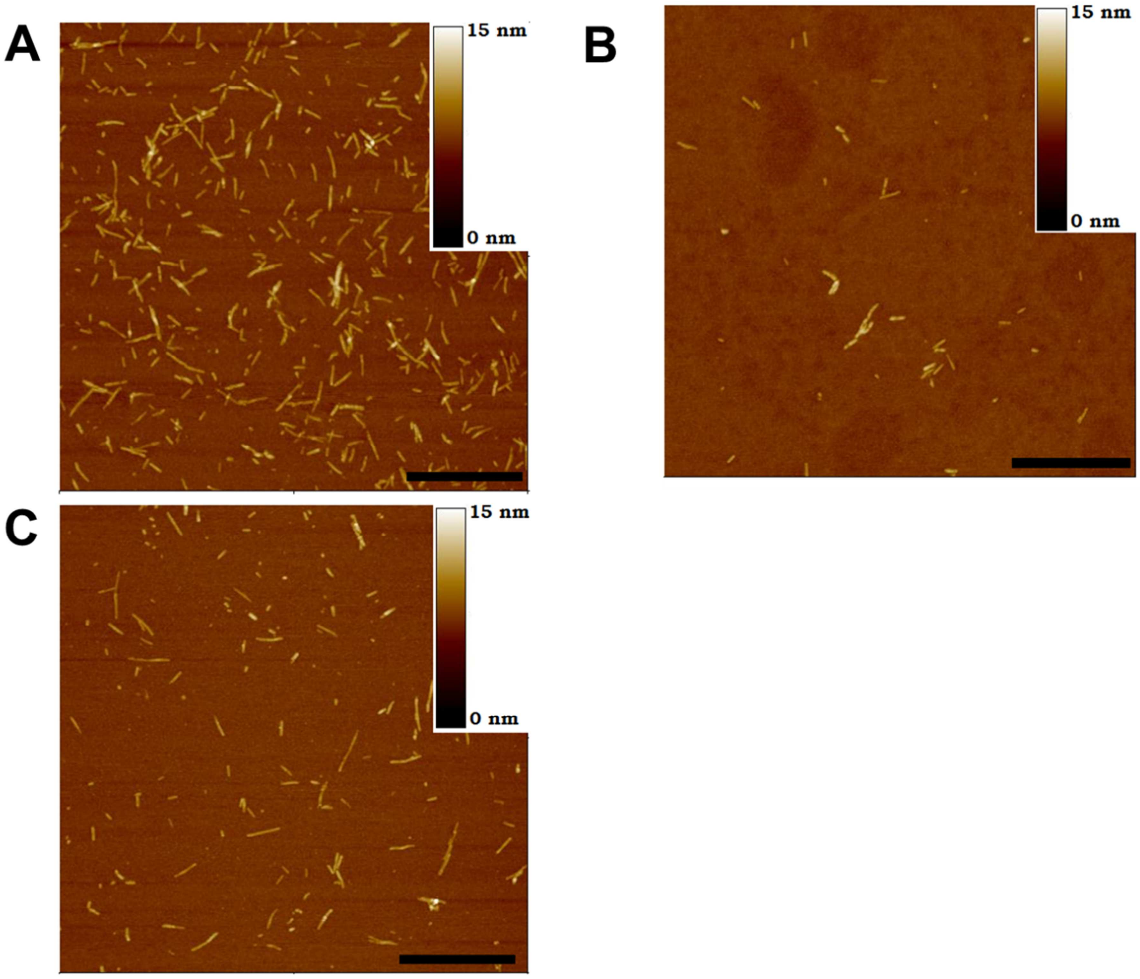

Supplementary Figure S3. AFM micrographs of a HEWL solution (0.2mM) previously incubated during 7d at pH 2.0 and 60°C in presence of 20mM of **(A)** 2HP, **(B)** 3HP or **(C)** 4HP. The scale bar represents 0.5μm.

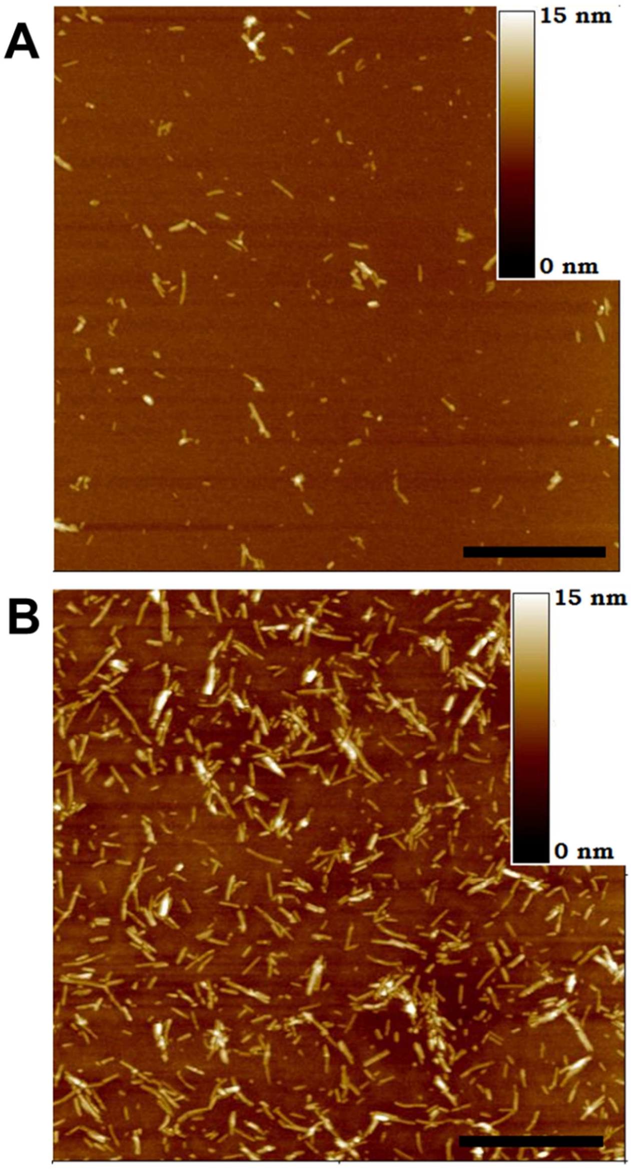

Supplementary Figure S4. AFM micrographs of a HEWL solution (0.2mM) previously incubated during 16d at pH 2.0 and 60°C in presence of 20mM of **(A)** 2Cl-3HP or **(B)** 5Cl-3HP. The scale bar represents 0.5µm.

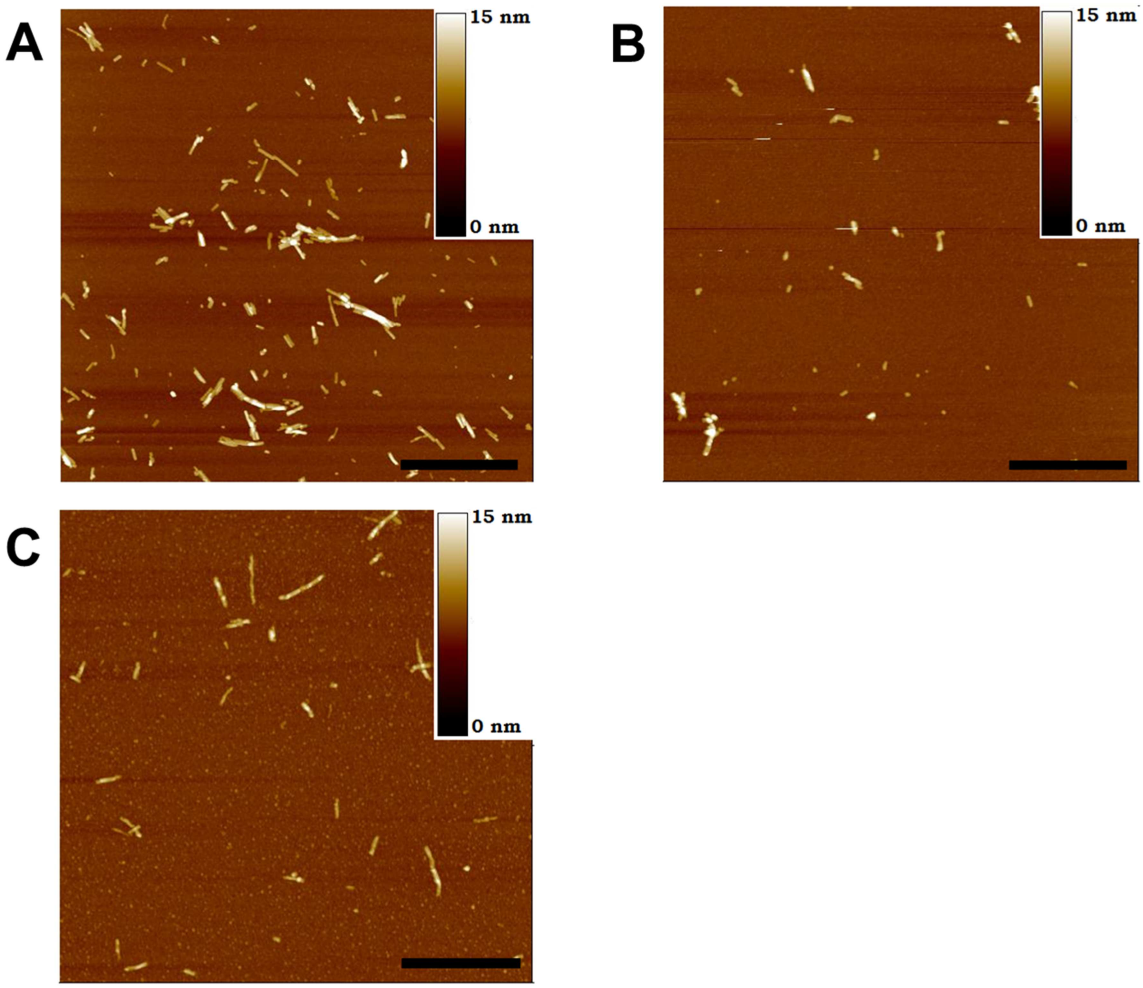

Supplementary Figure S5. AFM micrographs of a HEWL solution (0.2mM) previously incubated during 16d at pH 2.0 and 60°C in presence of 20mM of **(A)** PM, **(B)** PL or **(C)** PN. The scale bar represents 0.5μm.

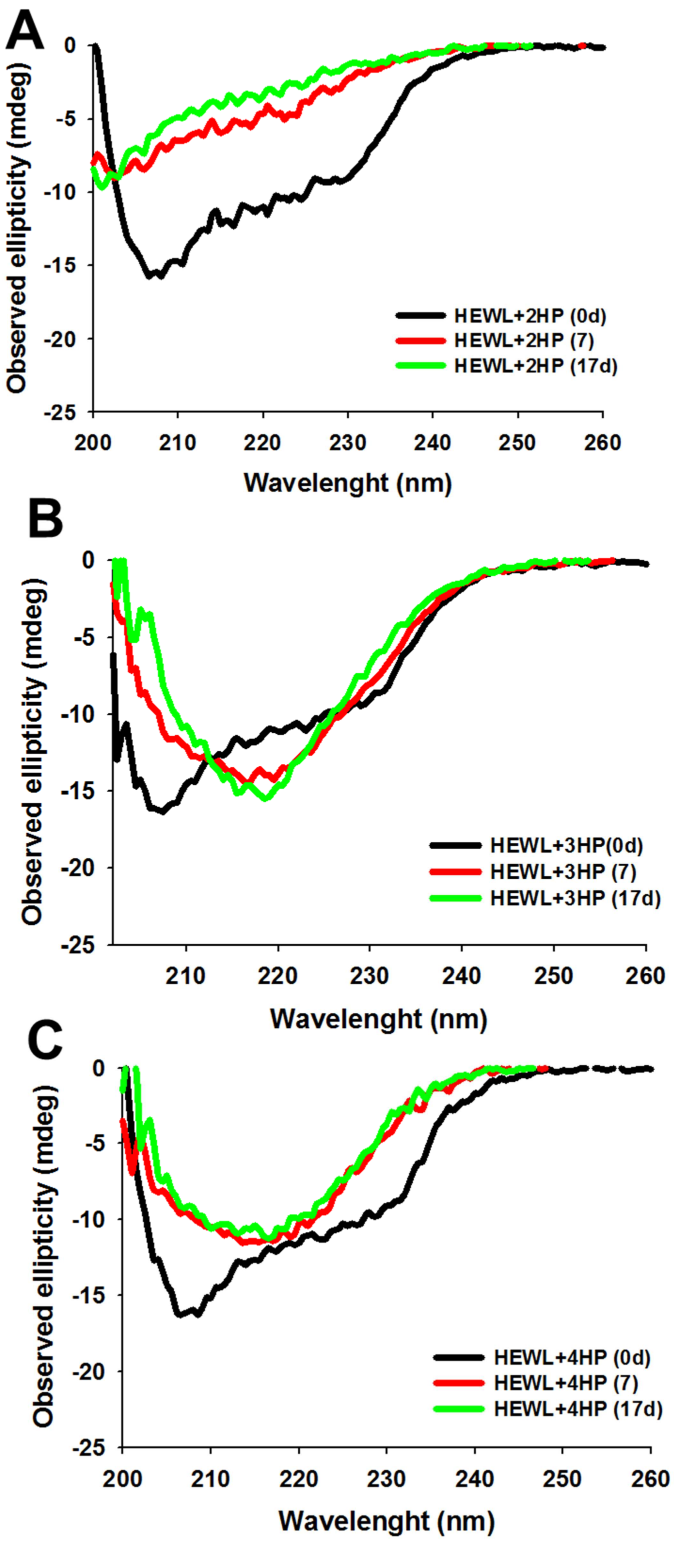

Supplementary Figure S6. Far-UV CD spectra of a HEWL sample incubated **(A)** in the presence of 20mM of 2HP, **(B)** in the presence of 20mM of 3HP or **(C)** in the presence of 20mM of 4HP at pH 2.0 and 60°C for 0, 7 and 17d.

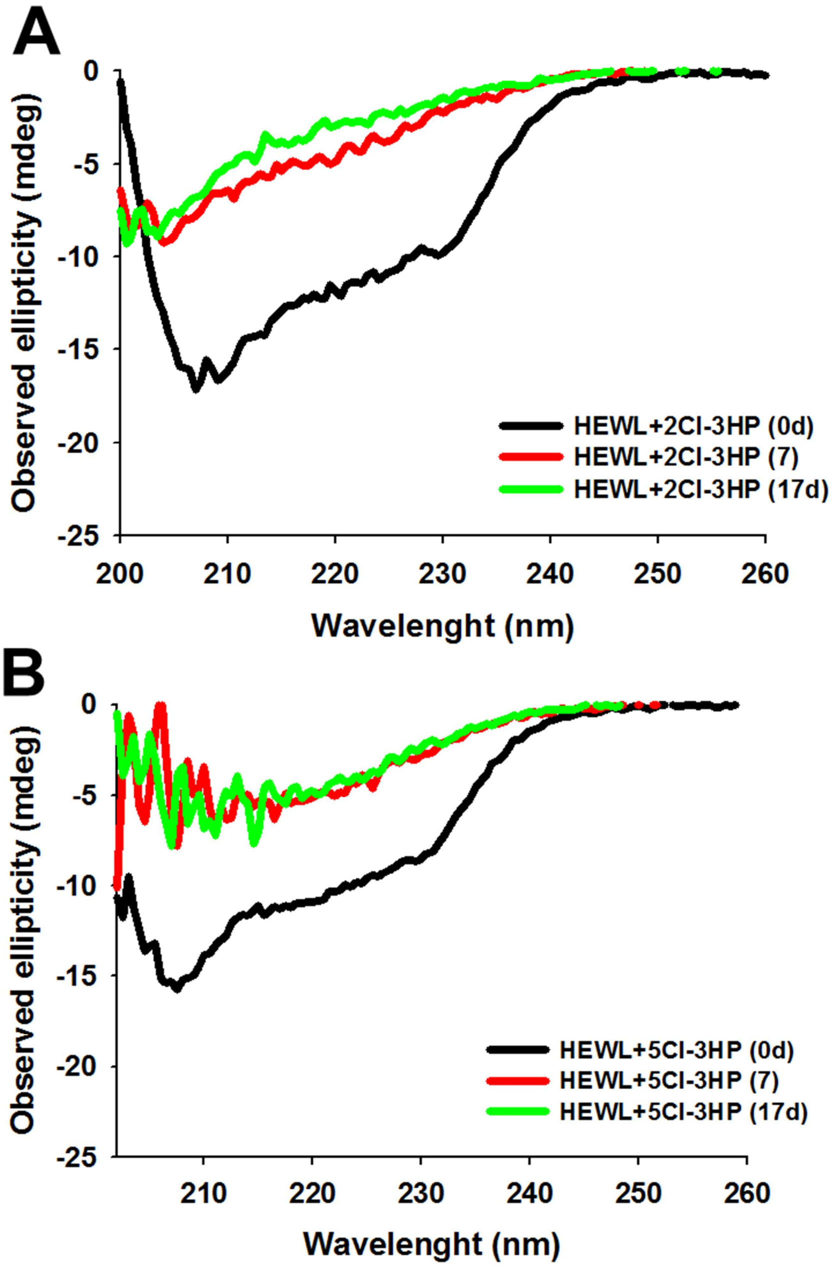

Supplementary Figure S7. Far-UV CD spectra of a HEWL sample incubated **(A)** in the presence of 20mM of 2Cl-3HP or **(B)** 5Cl-3HP at pH 2.0 and 60°C for 0, 7 and 17 d.

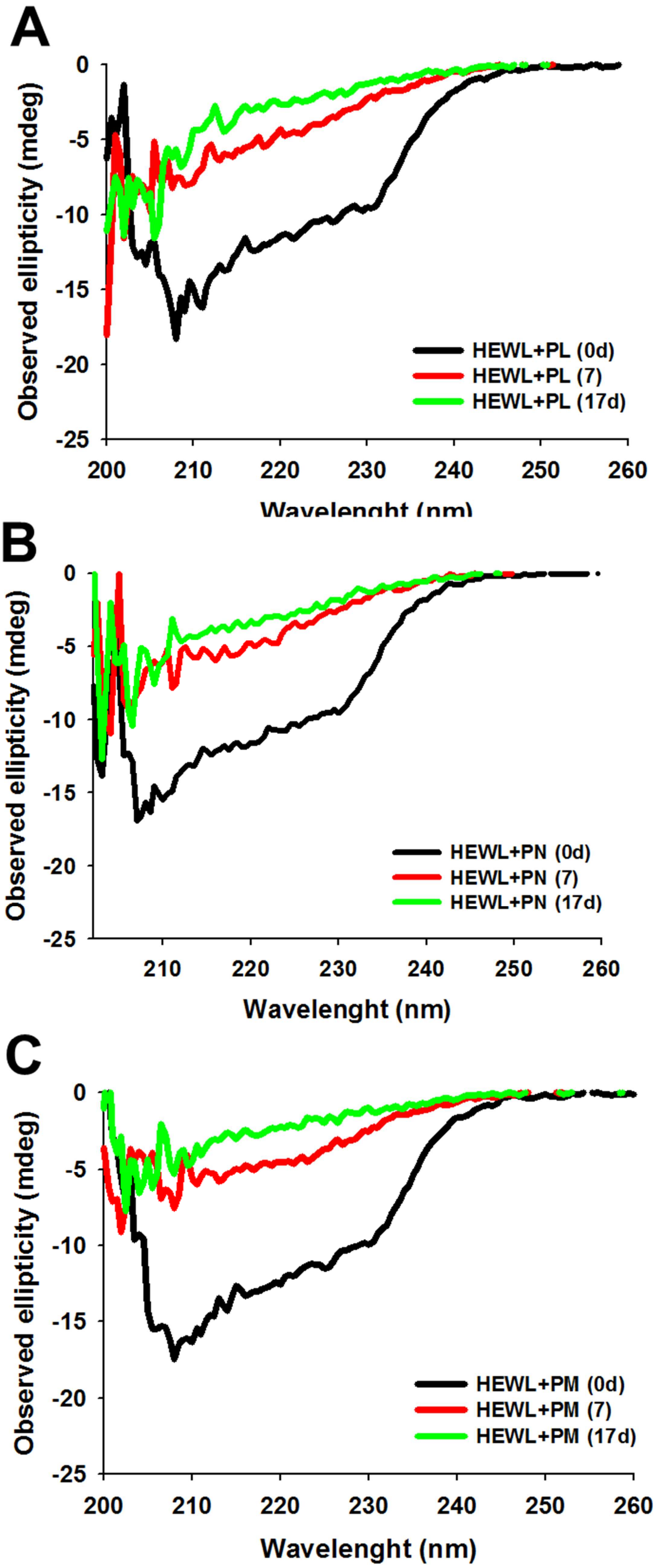

Supplementary Figure S8. Far-UV CD spectra of a HEWL sample incubated **(A)** in the presence of 20mM of PL, **(B)** PN or **(C)** PM at pH 2.0 and 60°C for 0, 7 and 17d.

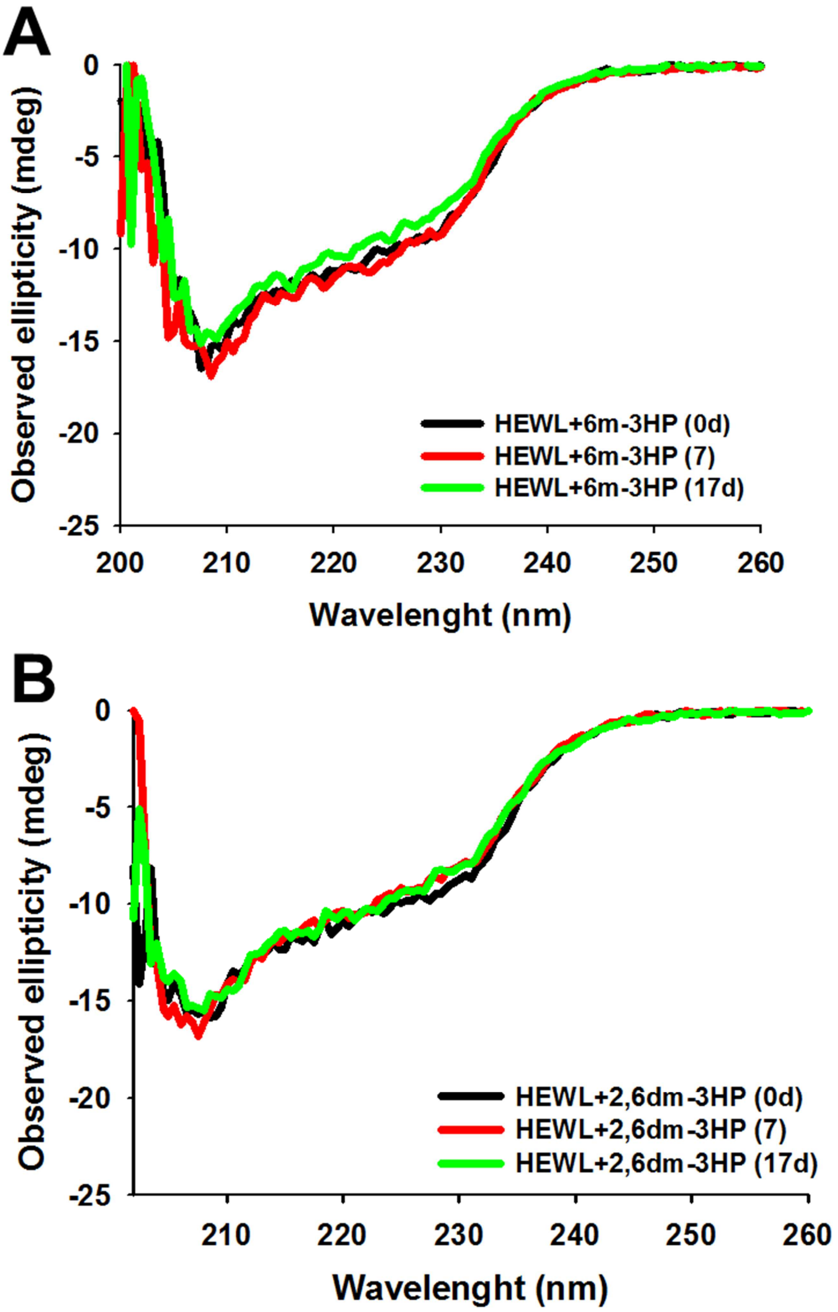

Supplementary Figure S9. Far-UV CD spectra of a HEWL sample incubated **(A)** in the presence of 20mM of 6m-3HP or **(B)** 2,6dm-3HP at pH 2.0 and 60°C for 0, 7 and 17d.

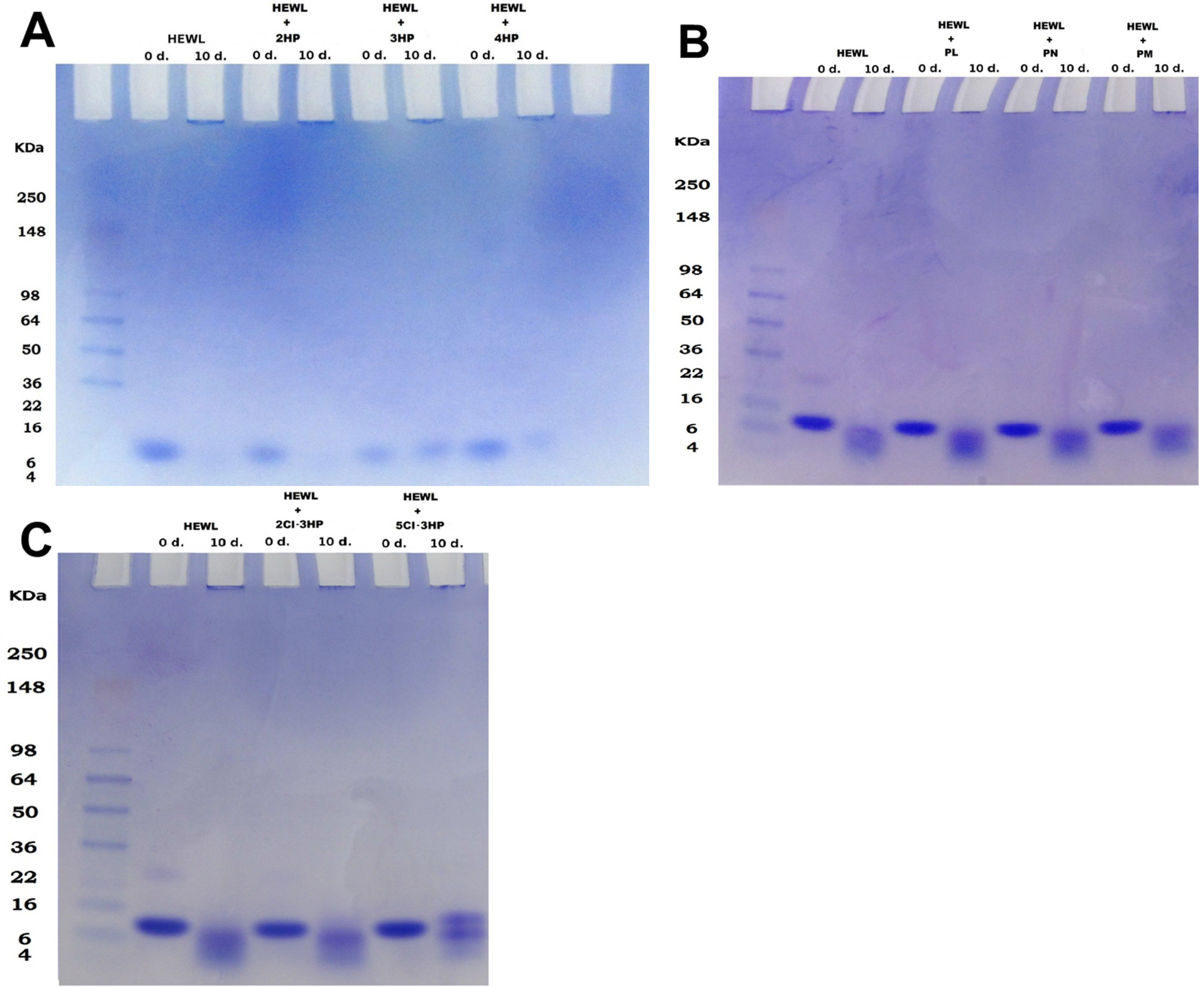

Supplementary Figure S10. SDS-PAGE gel analysis of solutions containing HEWL incubated at pH 2.0 and 60°C during 0 and 10d either alone or in the presence of **(A)** 2HP, 3HP or 4HP; **(B)** PL, PN or PM; **(C)** 2Cl-3HP or 5Cl-3HP.

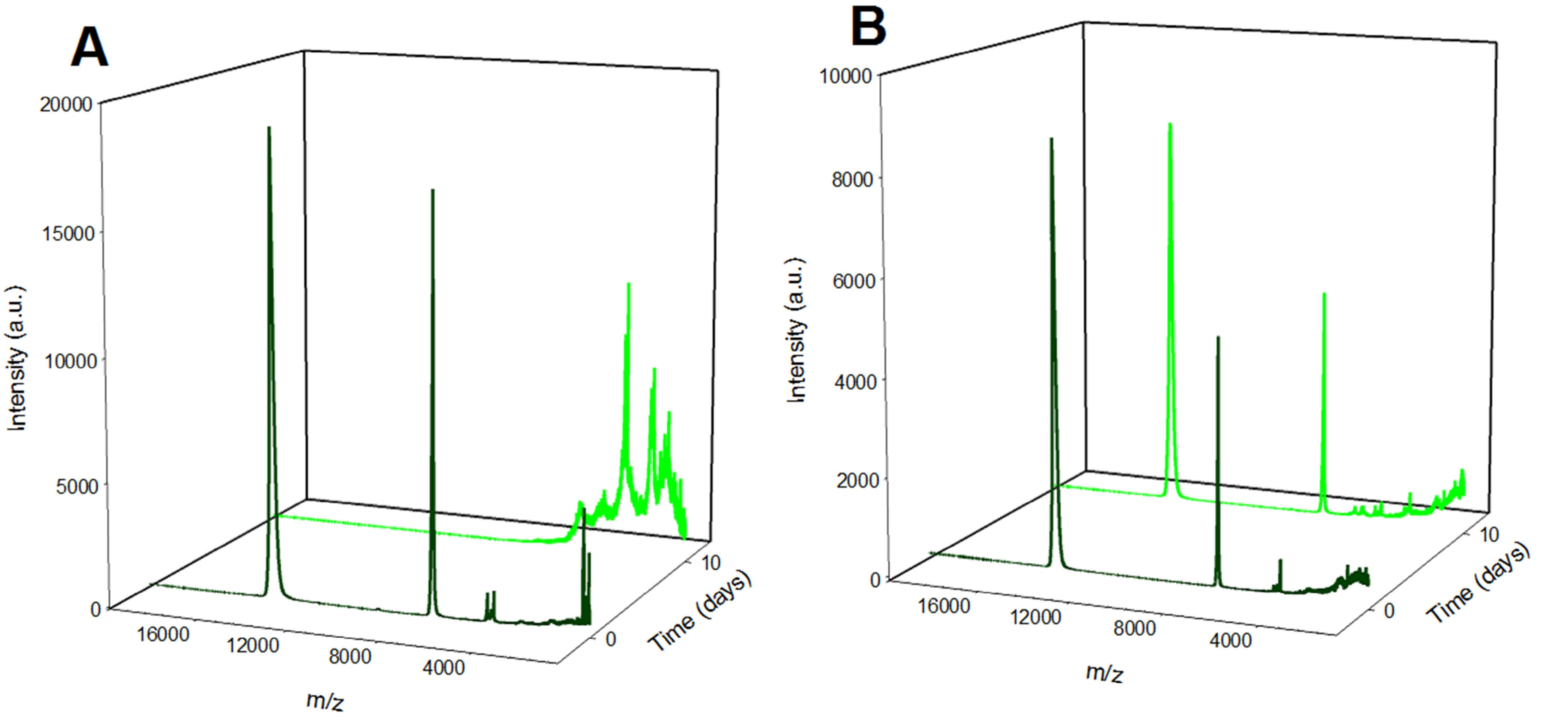

Supplementary Figure S11. Time-dependent MALDI-TOF/TOF spectra of a HEWL solution (0.2mM) incubated alone **(A)** or in the presence of 20mM 6m-3HP **(B)** at pH 2.0 and 60°C.

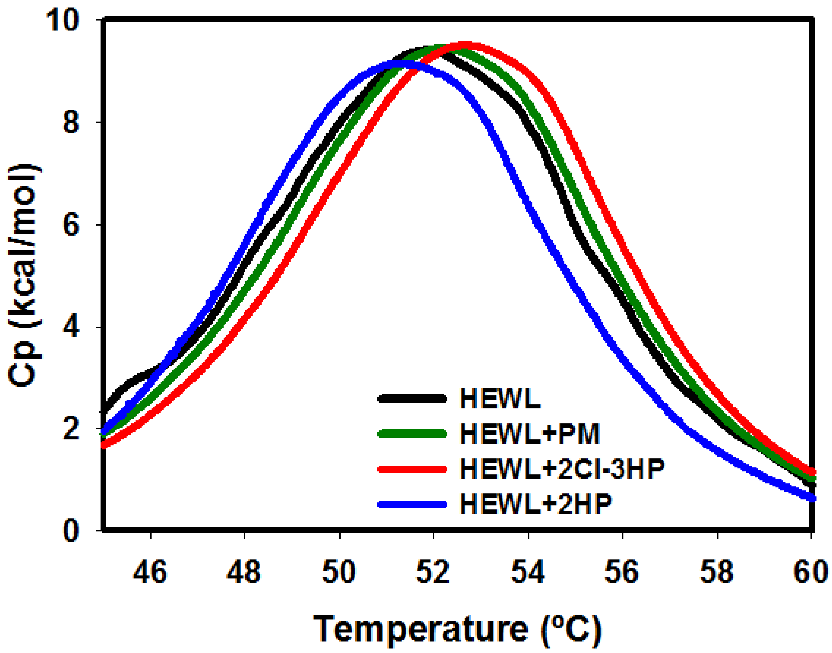

Supplementary Figure S12. Differential scanning calorimetry thermograms of different HEWL solutions (0.2mM) at pH 2.0 acquired in the absence (black curve) or in the presence of 20mM of three different HPs.

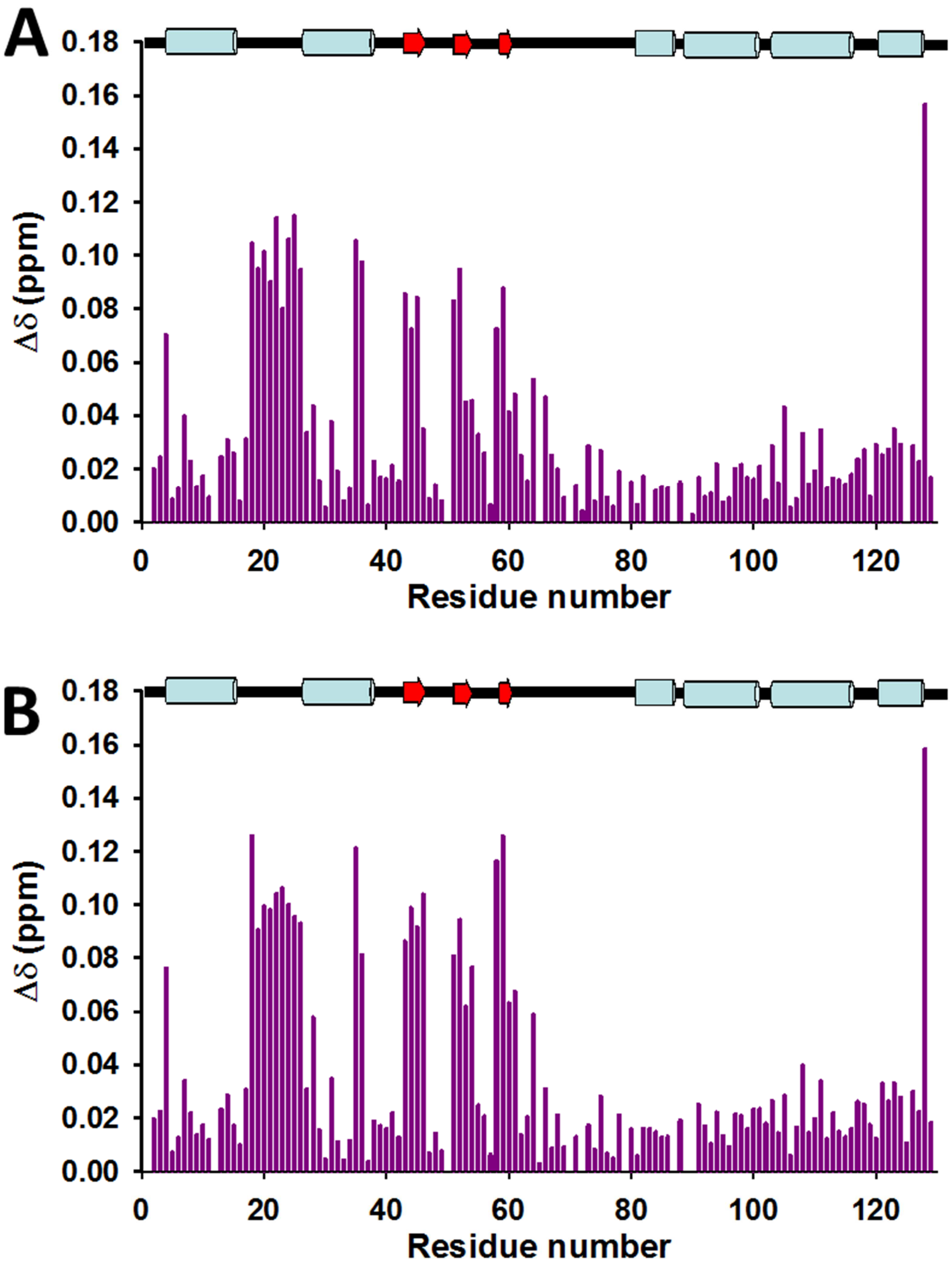

Supplementary Figure S13. Chemical shift histograms of the weighted amide chemical shift perturbation observed along the HEWL sequence after addition of **(A)** 6m-3HP or **(B)** 2,6dm-3HP at a HEWL:HP ratio of 1:5. The secondary structure is indicated on the plot as reference.

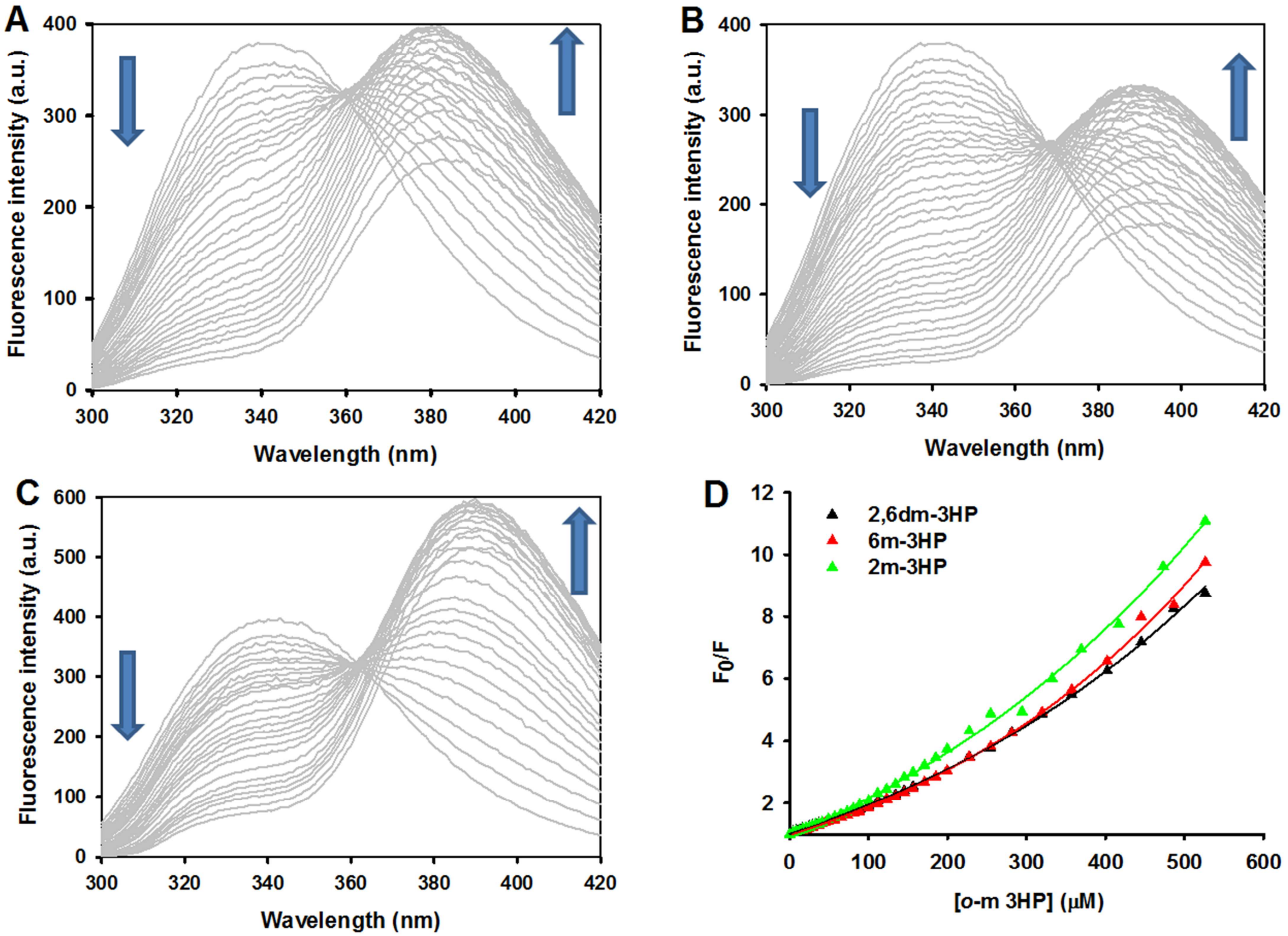

Supplementary Figure S14. Effect of *o*-methylated 3HPs on the fluorescent emission spectrum of HEWL. **(A-C)** Changes in the intrinsic fluorescent spectra of HEWL at 37°C (λ_{exc} 280nm) upon addition of different aliquots of solutions containing **(A)** 2m-3HP, **(B)** 6m-3HP or **(C)** 2,6dm-3HP, from 0 to 526μM concentration. **(D)** Stern-Volmer plot for solutions containing native HEWL. F and F_0 are the fluorescence intensities (λ_{exc} 280nm, λ_{em} 340nm) in the absence or presence of increasing concentrations of *o*-methylated 3HP.

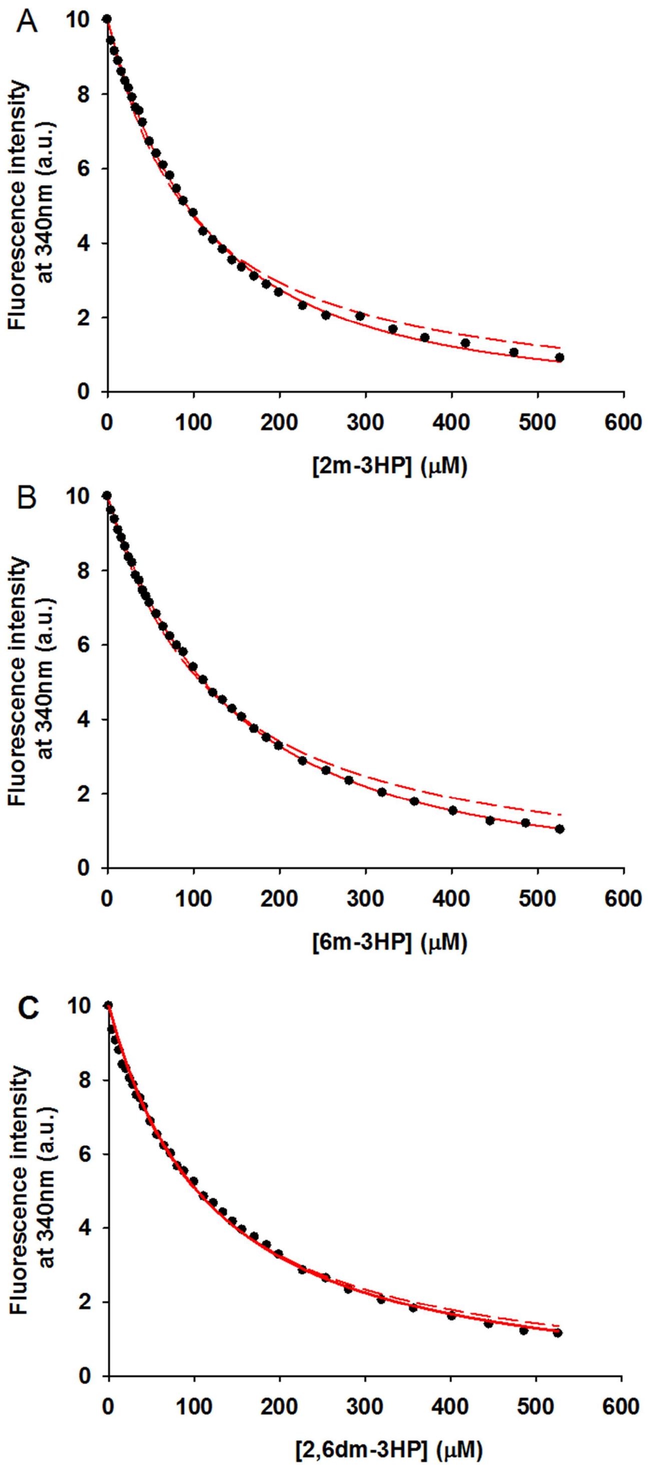

Supplementary Figure S15. Changes in the fluorescence intensity of HEWL measured at 340nm (λ_{exc} 280nm) as function of **(A)** 2m-3HP, **(B)** 6m-3HP or **(C)** 2,6dm-3HP concentrations at pH 2.0 and 37°C. Experimental data is shown as black dots. Dashed red lines (- - -) represent the best theoretical fit to a model involving one protein binding site, thus forming a 1:1 protein-HP complex. Continuous red lines (—) represent the best theoretical fit to a model involving two independent binding sites, thus forming a 1:2 protein-HP complex. The experimental data was fitted to both models using the Dynafit software taking into account the HEWL and the *o*-methylated 3HP dilution effects associated to the titration.

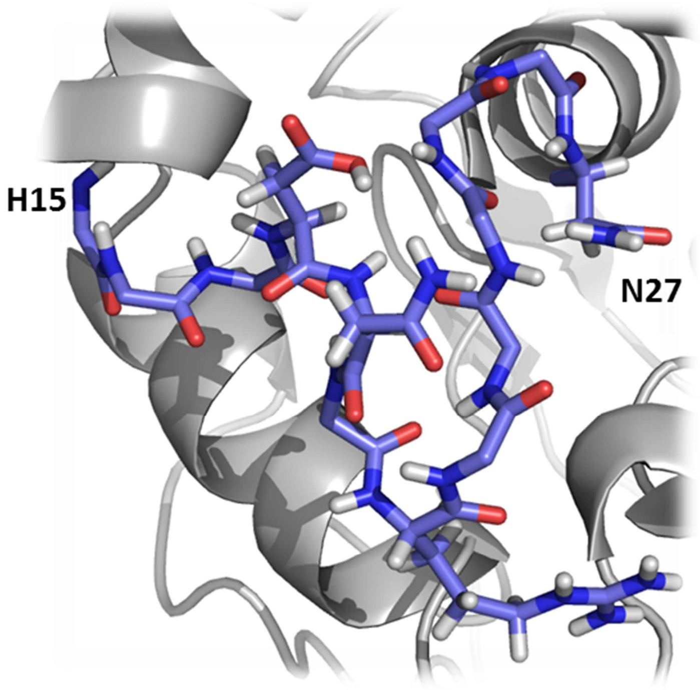

Supplementary Figure S16. Cartoon representation of HEWL (PDB code 1LSE). The entirely HEWL structure is shown as cartoons (grey), except the unstructured region between the helices A and B (H15-G26) which was colored in atom-coloured sticks. The side chains of the residues within this region capable to accept/donate hydrogen are also shown as atom-coloured sticks (D18, N19, R21 and N27). The image was generated using the molecular graphics software PyMOL.

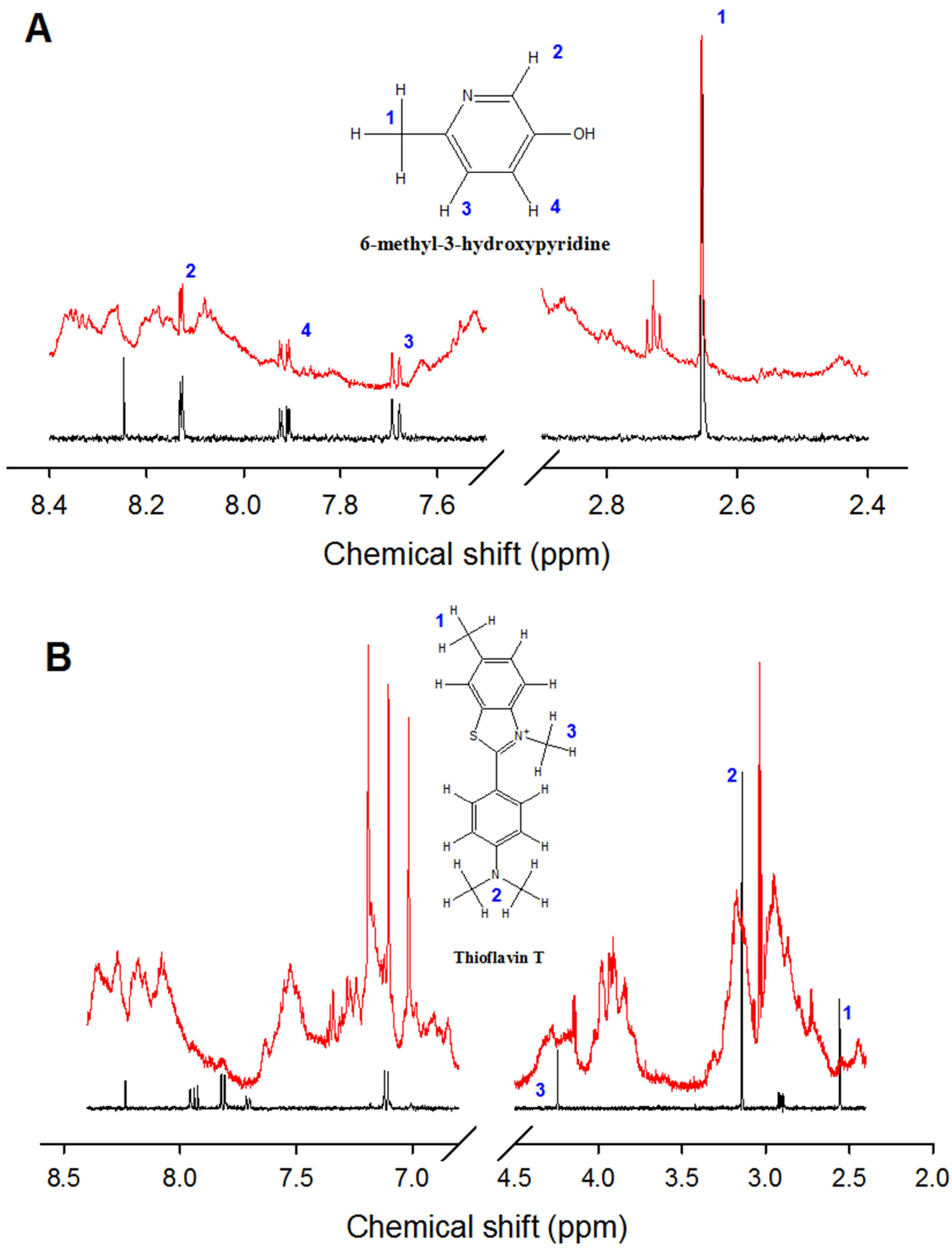

Supplementary Figure S17. Superposition of the ^{1}H-NMR spectra of **(A)** 6m-3HP or **(B)** ThT acquired alone (*black*) and upon addition of preformed HEWL amyloid fibrils (*red*). All NMR spectra were collected in presence of 137mM NaCl and 2.6mM KCl at pH 2.0 and at 25°C.

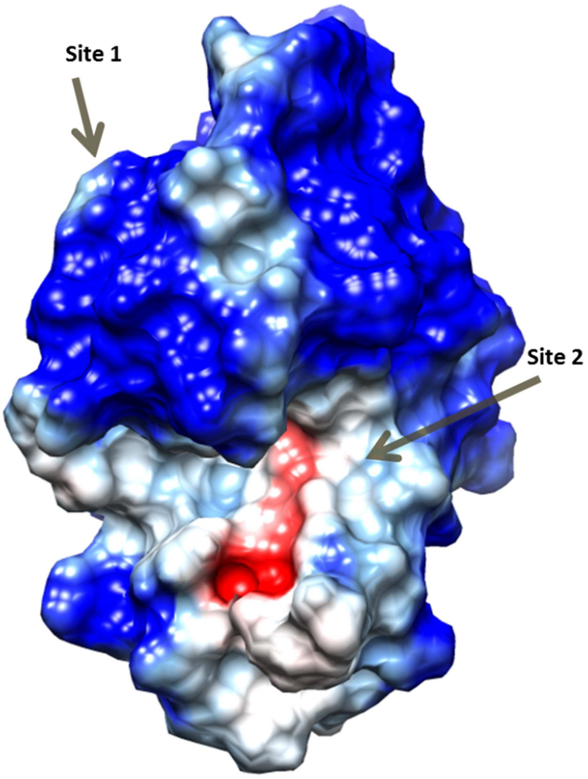

Supplementary Figure S18. Surface of the electrostatic molecular potential of HEWL (PDB code 1LSE) at pH 2.0. The colors *red* to *blue* represent the neutral (0) and the positive (5) potential measured in kT/e by using Chimera software.

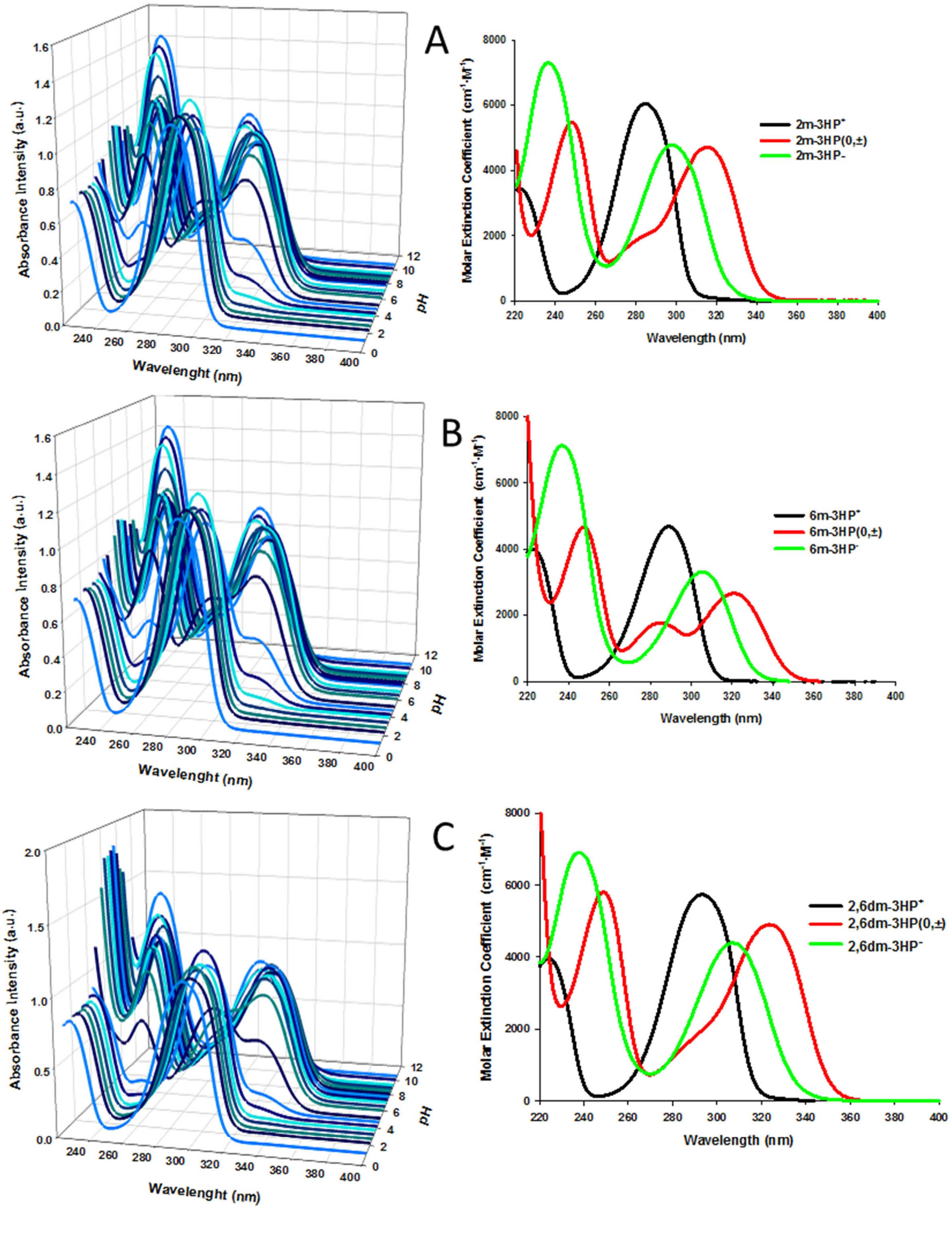
A
Absorbance Intensity (a.u.)
Wavelenght (nm)
pH
Molar Extinction Coefficient (cm-1·M-1)
Wavelength (nm)
2m-3HP+
2m-3HP(0,±)
2m-3HP-
B
Absorbance Intensity (a.u.)
Wavelenght (nm)
pH
Molar Extinction Coefficient (cm-1·M-1)
Wavelength (nm)
6m-3HP+
6m-3HP(0,±)
6m-3HP-
C
Absorbance Intensity (a.u.)
Wavelenght (nm)
pH
Molar Extinction Coefficient (cm-1·M-1)
Wavelength (nm)
2,6dm-3HP+
2,6dm-3HP(0,±)
2,6dm-3HP-

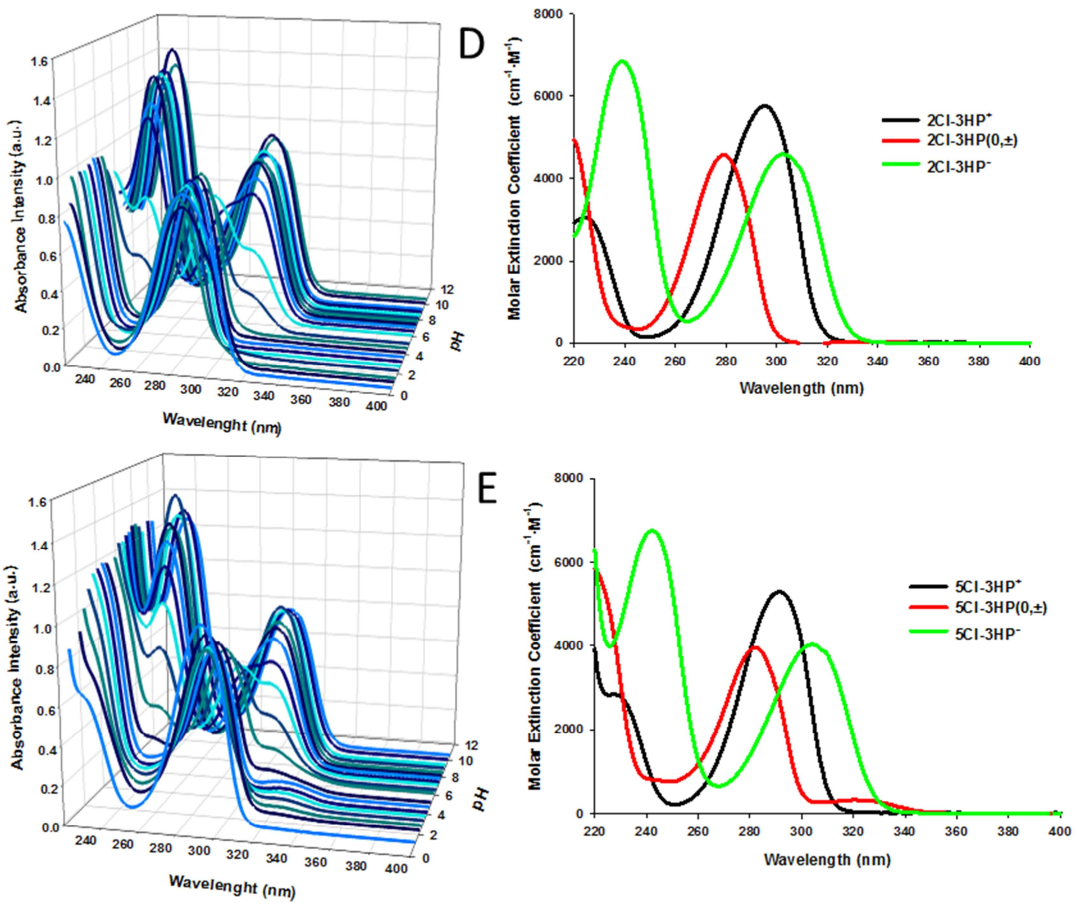

Supplementary Figure S19. UV-Vis spectra of 2m-3HP (**A**), 6m-3HP (**B**), 2,6m-3HP (**C**), 2Cl-3HP (**D**) and 5Cl-3HP (**E**) at different pH (*left panels).* UV-Vis spectra of cationic, neutral and anionic forms of 2m-3HP (**A**), 6m-3HP (**B**), 2,6m-3HP (**C**), 2Cl-3HP (**D**) and 5Cl-3HP (**E**) obtained using SEPCFIT/32™ analysis software from 20 individual UV-Vis spectra at pH between 1 and 13 (*right panels).*

```
[task]

   task = fit
   data = equilibria

[mechanism]

   P + L <==> PL    :    K1 equil
   PL + L <==> PLS :    K2 equil

[constants]

   K1 = .010 ?, K2 = .004 ?

[concentrations]
   P = 10
   L  = 0

[equil]

   variable L, P
   plot titration
   file ./HP/2m-3HP/data/data.txt
   response P = 1

[output]

   directory ./HP/2m-3HP/output

[end]
```

Supplementary Figure S20. DynaFit script file that was used to fit the changes in the fluorescence intensity of HEWL as function of *o*-methylated 3HPs concentration to a model involving two independent binding sites. The experimental data was fitted to this multivariable model taking into account the simultaneous variation of the *o*-methylated 3HPs and the HEWL concentrations at each titration point. Therefore the "*data.txt*" file contained three different columns. The first one included the variation of the ligand concentration at each titration point. The second one contained the variation in the HEWL concentration at each titration point. The third included the variation of the fluorescence intensity at each titration point.

Supporting Tables

Supplementary Table S1. Secondary structure content of HEWL at different incubation times in the absence and in the presence of different HPs.

	% Helix[a]			% β-sheet[a]			% Turns[a]			% Random Coil[a]		
**Days**	**0**	**7**	**17**	**0**	**7**	**17**	**0**	**7**	**17**	**0**	**7**	**17**
**HEWL**	34.8	8.9	7.4	7.1	28.5	32.2	14.1	14.5	13.9	44.1	48.1	46.5
**PDB: 1E8L[b]**	*32.6*			*0.0*			*20.2*			*47.3*		
**PDB: 1GXV[b]**	*30.2*			*7.0*			*22.5*			*40.3*		
**HEWL+2HP**	34.3	7.7	4.8	3.9	28.7	26.1	14.8	15.6	16.4	46.9	48.1	52.7
**HEWL+3HP**	33.6	14.9	8.2	4.8	32.2	34.6	13.3	8.8	5.5	48.3	44.1	51.7
**HEWL+4HP**	33.4	14.2	12.0	7.2	42.6	17.4	12.9	9.0	17.5	46.5	34.2	53.1
**HEWL+2Cl-3HP**	32.7	10.1	4.6	9.8	24.2	29.8	15.5	15.6	16.1	42.0	50.2	49.5
**HEWL+5Cl-3HP**	29.6	4.6	12.5	8.1	36.6	38.5	14.6	13.9	12.5	47.7	44.9	36.5
**HEWL+2m-3HP**	33.9	32.3	34.0	23.3	14.8	12.4	14.7	15.4	15.8	28.1	37.5	37.7
**HEWL+6m-3HP**	33.2	34.7	32.4	25.2	23.0	24.1	16.2	14.5	13.0	25.3	27.5	30.5
**HEWL+2,6dm-3HP**	32.7	35.4	25.9	18.6	22.0	24.3	11.3	15.0	11.7	37.4	27.6	38.2
**HEWL+PM**	33.0	4.3	7.4	20.9	27.1	32.2	12.6	15.7	13.9	33.5	53.0	46.5
**HEWL+PN**	31.7	1.6	2.0	9.6	39.6	39.4	11.2	18.7	19.2	47.4	40.1	39.4
**HEWL+PL**	31.2	1.2	0.9	21.7	31.4	23.7	14.8	20.3	19.5	32.2	47.2	55.9

[a]The secondary structure contents were estimated by using the BeStSel on-line platform (http://bestsel.elte.hu/)
[b]The secondary structure contents for the PDB entries of HEWL were derived using the BeStSel platform.

Supplementary Table S2. Molecular docking results of HEWL with different *o*-methylated 3HPs at the binding sites 1 and 2.

	Site 1			*Site 2*		
	**Autodock Binding Energy (kcal/mol)[a]**	**Interacting HEWL residues**	**Ligand RMS (Å)[b]**	**Autodock Binding Energy (kcal/mol)[a]**	**Interacting HEWL residues**	**Ligand RMS (Å)[b]**
**2m-3HP**	-2.8±0.1	K13[c], D18[c], L25[c], Q121[c]	0.06±0.07[d]<br>19.0±20.6[e]	-3.6±0.1	E35[c], N44[c], R45[c], D52[c]	1.76±2.03[f]
**6m-3HP**	-3.0±0.1	D18[c], N19[c], Y20, R21[c]	0.03±0.10	-4.1±0.1	E35[c], N44[c], D52	0.11±0.12
**2,6dm-3HP**	-3.0±0.1	G16[c], D18[c]	0.17±0.09[g]<br>0.08±0.08[h]	-3.9±0.1	E35[c], N44[c], R45[c], N46	0.10±0.06

[a]Averaged energy obtained from the fifteen conformations with lower energy.
[b]The RMS values were obtained from the different fifteen conformations taking lowest energy conformation as reference.
[c]The interaction between these residues and the ligand occurs through a hydrogen bond.
[d]The RMS value render the variation between the structures ensemble in the cluster shown in white in **Figure 5a *(left).***
[e]The RMS value render the variation between the two clustered ensembles (green and brown ligands) shown in the **Figure 5a *(left).***
[f]The RMS value implies the variation between the two clustered ensembles found for this binding site.
[g]The RMS value render the variation between the structures ensemble in the cluster shown in white in **Figure 5c *(left).***
[h]The RMS value render the variation between the structures ensemble in the cluster shown in green in **Figure 5c *(left).***

Supplementary Table S3. Mulliken charges for the different HPs computed at SMD-M06-2X/6-311++G(d,p) level of theory.

HP	q(O)[a]	q(oH)[b]	q(OH)[c]	q(N)[d]	q(nH)[e]	q(NH)[f]	q(Cl)[g]
**2,6dm-3HP**	-0.58	0.47	-0.11	-0.19	0.44	0.25	
**2m-3HP**	-0.57	0.47	-0.10	-0.18	0.45	0.27	
**6m-3HP**	-0.57	0.46	-0.11	-0.18	0.45	0.27	
**2HP**	-0.74	--[h]	-0.74	-0.37	0.42	0.05	
**3HP**	-0.56	0.46	-0.10	-0.22	0.46	0.24	
**4HP**	-0.52	0.46	-0.06	-0.21	0.45	0.23	
**5Cl-3HP**	-0.55	0.46	-0.08	-0.21	0.47	0.26	0.18
**2Cl-3HP**	-0.56	--[h]	-0.10	-0.11	0.48	0.37	0.14
**PL**	-0.58	0.48	-0.10	-0.20	0.46	0.26	
**PM**	-0.57	0.47	-0.09	-0.16	0.45	0.29	
**PN**	-0.60	0.50	-0.11	-0.19	0.45	0.26	

[a]Atomic charge corresponding to the phenolic oxygen.
[b]Atomic charge corresponding to the phenolic hydrogen.
[C]Total charge of the phenol group (O+H).
[d]Atomic charge corresponding to the pyridinic nitrogen.
[e]Atomic charge corresponding to the pyridinic hydrogen.
[f]Total charge of the pyridinic group (N+H)
[g]Atomic charge of the chlorine atom.
[h]The main ionic form of these compounds at pH 2.0 exhibits their pyridinic nitrogen unprotonated.

Supplementary Table S4. NBO charges of different HPs computed at SMD-M06-2X/6-311++G(d,p) level of theory.

HP	q(O)[a]	q(oH)[b]	q(OH)[c]	q(N)[d]	q(nH)[e]	q(NH)[f]	q(Cl)[g]
**2,6dm-3HP**	-0.73	0.56	-0.17	-0.47	0.50	0.03	
**2m-3HP**	-0.73	0.56	-0.16	-0.46	0.51	0.05	
**6m-3HP**	-0.73	0.56	-0.17	-0.46	0.51	0.05	
**2HP**	-0.80	--[h]	-0.80	-0.59	0.49	-0.10	
**3HP**	-0.73	0.56	-0.16	-0.46	0.52	0.06	
**4HP**	-0.70	0.57	-0.13	-0.50	0.51	0.01	
**5Cl-3HP**	-0.72	0.57	-0.15	-0.44	0.52	0.08	0.06
**2Cl-3HP**	-0.71	--[h]	-0.14	-0.48	0.53	0.05	0.11
**PL**	-0.72	0.57	-0.15	-0.46	0.51	0.06	
**PM**	-0.72	0.57	-0.15	0.52	-0.45	0.06	
**PN**	-0.74	0.56	-0.18	-0.46	0.51	0.05	

[a]Atomic charge corresponding to the phenolic oxygen.
[b]Atomic charge corresponding to the phenolic hydrogen.
[C]Total charge of the phenol group (O+H).
[d]Atomic charge corresponding to the pyridinic nitrogen.
[e]Atomic charge corresponding to the pyridinic hydrogen.
[f]Total charge of the pyridinic group (N+H)
[g]Atomic charge of the chlorine atom.
[h]The main ionic form of these compounds at pH 2.0 exhibits their pyridinic nitrogen unprotonated.

Supplementary Table S5. Charges from electrostatic potential of different HPs using Merz-Singh-Kollman (MK) Scheme computed at SMD-M06-2X/6-311++G(d,p) level of theory.

HP	q(O)[a]	q(oH)[b]	q(OH)[c]	q(N)[d]	q(nH)[e]	q(NH)[f]	q(Cl)[g]
**2,6dm-3HP**	-0.66	0.52	-0.14	-0.41	0.45	0.04	
**2m-3HP**	-0.65	0.52	-0.13	-0.27	0.43	0.16	
**6m-3HP**	-0.68	0.53	-0.16	-0.28	0.42	0.14	
**2HP**	-0.83	--[h]	-0.83	-0.32	0.35	0.03	
**3HP**	-0.69	0.53	-0.16	-0.10	0.39	0.28	
**4HP**	-0.68	0.53	-0.15	-0.21	0.39	0.17	
**5Cl-3HP**	-0.66	0.53	-0.13	-0.21	0.43	0.23	-0.03
**2Cl-3HP**	-0.70	--[h]	-0.16	0.04	0.35	0.38	0.04
**PL**	-0.61	0.50	-0.12	-0.17	0.42	0.26	
**PM**	-0.58	0.50	-0.09	-0.24	0.43	0.19	
**PN**	-0.67	0.52	-0.15	-0.39	0.47	0.08	

[a]Atomic charge corresponding to the phenolic oxygen.
[b]Atomic charge corresponding to the phenolic hydrogen.
[c]Total charge of the phenol group (O+H).
[d]Atomic charge corresponding to the pyridinic nitrogen.
[e]Atomic charge corresponding to the pyridinic hydrogen.
[f]Total charge of the pyridinic group (N+H)
[g]Atomic charge of the chlorine atom.
[h]The main ionic form of these compounds at pH 2.0 exhibits their pyridinic nitrogen unprotonated.

Supplementary Table S6. Computed electric dipole moments for different HPs.

HPs[a]	Electric Dipole Moment (*D*)[b]
**2HP**	7.40
**3HP**	3.45
**4HP**	4.84
**2Cl-3HP**	6.67
**5Cl-3HP**	5.33
**2m-3HP**	3.42
**6m-3HP**	2.26
**2,6dm-3HP**	2.37
**PM**	3.87
**PN**	5.85
**PL**	6.39

[a]The electric dipole moment calculation was carried out on the main ionic form present at pH 2.0, which was always the cationic one except for 2HP and 2Cl-3HP according their low pKa value (**Fig. 1**).
[b]Computed for the geometrically optimized structures at SMD-M06-2X/6-311++G(d,p) level of theory.

Supporting Methods

Chemicals and reagents

Hen-egg white lysozyme (HEWL) (EC 3.2.1.17), pyridoxamine (PM), 2-chloro-3-hydroxypyridine (2Cl-3HP), thioflavin T (ThT), pyridoxal (PL), pyridoxine (PN) and 3-(trimethylsilyl)-1-propanesulphonic acid (DSS), were purchased from Sigma-Aldrich. 2-hydroxypyridine (2HP), 3-hydroxypyridine (3HP), 4-hydroxypyridine (4HP), 3-hydroxy-2-methylpyridine (2m-3HP) and 3-hydroxy-6-methylpyridine (6m-3HP) were acquired from Acros Organics. 5-chloro-3-hydroxypyridine (5Cl-3HP) and 3-hydroxy-2,6-dimethylpyridine (2,6dm-3HP) were supplied by Mybridge. Buffer reagents were ACS grade. All solutions were prepared by using milli-Q water.

MALDI-TOF/TOF mass spectrometry

0.5µl aliquots of solutions containing HEWL (0.2mM) previously incubated during 0 or 10d at pH 2.0 and 60°C either alone or in the presence of 20mM 2m-3HP, 6m-3HP or 2,6dm-3HP were spotted onto a steel target plate (MTP 384), air-dried, and subjected to mass determination. Mass spectra were analyzed on a Bruker Autoflex III MALDI-TOF spectrometer equipped with a 200-Hz smart-beam pulsed N2 laser (λ 337 nm). The IS1 and IS2 voltages were 19 kV and 16.65 kV respectively, and the lens voltage was 8.2 kV. Measurements were performed using a positive reflector mode with matrix suppression below 400 Da. External calibration was performed using a standard peptide mixture. Mass spectra of digested samples were matched against Swiss-Prot databases using the Mascot search engine (Matrix Sciences).

Determination of the ionization equilibrium constants

The ionization equilibrium constants for 2m-3HP, 6m-3HP, 2,6dm-3HP, 2Cl-3HP and 5Cl-3HP were determined acquiring the corresponding absorption spectra at different pH using a Shimadzu UV-2401 PC double-beam spectrophotometer thermostated at 37°C. Quartz cells of 1-cm path length were used to obtain the electronic spectra. Spectroscopic data were acquired over the range from 500 to 220nm. The buffer solution background spectrum was used as spectral reference. UV-Vis spectra were obtained in various 10mM buffers having a constant ionic strength of 0.5M that was adjusted by addition of KCl. The reagents used to prepare the buffer solutions were HCl (pH 1), sodium chloroacetate (pHs 2, 2.5, 3 and 3.5), sodium acetate (pHs 4, 4.5 and 5), succinic acid (pHs 6 and 6.5), potassium dihydrogen phosphate (pHs 7, 7.5 and 8), boric acid (pHs 8.5, 9 and 9.5), potassium bicarbonate (pHs 10 and 11) and NaOH (pHs 12 and 13). Each buffer was used to prepare a solution containing 0.2mM of each different 3HP. The individual UV-Vis spectra obtained at each pH were used as inputs for the factor analysis software SEPCFIT/32™. This allowed to obtain the characteristic UV-Vis spectrum for each ionic form and its macroscopic ionization constants.

Ab-initio calculation of electronic dipolar moment of different HPs

Standard density functional theory (DFT) calculations were carried out with the Gaussian09 software[1]. The structures corresponding to the ionic forms with a higher population at pH 2.0 were optimized for each studied HP by using the M06-2X functional in combination with the 6-311++G(d,p) basis set. Vibrational analyses were performed to verify all the optimized structures as energy minima by the absence of imaginary frequencies. All the calculations were carried out in aqueous solution modeled by the SMD solvent model[2]. The dipole moments of all HPs were calculated from the wave functions of the optimized structures in aqueous solution. In addition, wave functions were used to compute Merz-Kollman charges, Mulliken charges and the natural bond orbital charges.

Statistical analysis

The correlation between the dipolar moments computed for the main ionic forms of each HP at pH 2.0, and the fibril growth rate constant (k_f) or the fibril lag-phase, were analyzed using linear regression models and Pearson correlation coefficients. A two-tailed p value < 0.05 was considered statistically significant. Statistical analyses were performed using SPSS 19.0 software (SPSS Inc., Chicago, IL, USA).

Supporting References

1. Frisch, M. J. et al. Gaussian 09 Revision C.01, Gaussian, Inc., Wallingford, CT, 2010.
2. Marenich, A. V., Cramer, C. J. & Truhlar, D. G. Universal solvation model based on solute electron density and on a continuum model of the solvent defined by the bulk dielectric constant and atomic surface tensions. *J. Phys. Chem. B* **113**, 6378-6396 (2009).
